# A systematic review of biomarkers multivariately associated with acute respiratory distress syndrome development and mortality

**DOI:** 10.1186/s13054-020-02913-7

**Published:** 2020-05-24

**Authors:** Philip van der Zee, Wim Rietdijk, Peter Somhorst, Henrik Endeman, Diederik Gommers

**Affiliations:** Department of Adult Intensive Care, Erasmus Medical Center Rotterdam, Dr. Molewaterplein 40, 3015 GD Rotterdam, The Netherlands

**Keywords:** Acute respiratory distress syndrome, Biomarkers, Diagnosis, Mortality

## Abstract

**Background:**

Heterogeneity of acute respiratory distress syndrome (ARDS) could be reduced by identification of biomarker-based phenotypes. The set of ARDS biomarkers to prospectively define these phenotypes remains to be established.

**Objective:**

To provide an overview of the biomarkers that were multivariately associated with ARDS development or mortality.

**Data sources:**

We performed a systematic search in Embase, MEDLINE, Web of Science, Cochrane CENTRAL, and Google Scholar from inception until 6 March 2020.

**Study selection:**

Studies assessing biomarkers for ARDS development in critically ill patients at risk for ARDS and mortality due to ARDS adjusted in multivariate analyses were included.

**Data extraction and synthesis:**

We included 35 studies for ARDS development (10,667 patients at risk for ARDS) and 53 for ARDS mortality (15,344 patients with ARDS). These studies were too heterogeneous to be used in a meta-analysis, as time until outcome and the variables used in the multivariate analyses varied widely between studies. After qualitative inspection, high plasma levels of angiopoeitin-2 and receptor for advanced glycation end products (RAGE) were associated with an increased risk of ARDS development. None of the biomarkers (plasma angiopoeitin-2, C-reactive protein, interleukin-8, RAGE, surfactant protein D, and Von Willebrand factor) was clearly associated with mortality.

**Conclusions:**

Biomarker data reporting and variables used in multivariate analyses differed greatly between studies. Angiopoeitin-2 and RAGE in plasma were positively associated with increased risk of ARDS development. None of the biomarkers independently predicted mortality. Therefore, we suggested to structurally investigate a combination of biomarkers and clinical parameters in order to find more homogeneous ARDS phenotypes.

**PROSPERO identifier:**

PROSPERO, CRD42017078957

## Introduction

The acute respiratory distress syndrome (ARDS) is a major problem in the intensive care unit (ICU) with a prevalence of 10% and an in-hospital mortality rate of 40% [1, 2]. ARDS pathophysiology is based on a triad of alveolar-capillary membrane injury, high permeability alveolar oedema, and migration of inflammatory cells [3]. This triad is not routinely measured in clinical practice. Therefore, arterial hypoxemia and bilateral opacities on chest imaging following various clinical insults are used as clinical surrogates in the American European Consensus Conference (AECC) definition and the newer Berlin definition of ARDS [4, 5].

Histologically, ARDS is characterized by diffuse alveolar damage (DAD). The correlation between a clinical and histological diagnosis of ARDS is poor [6]. Only half of clinically diagnosed patients with ARDS have histological signs of DAD at autopsy [7–10]. The number of risk factors for ARDS and consequently the heterogeneous histological substrates found in patients with clinical ARDS have been recognized as a major contributor to the negative randomized controlled trial results among patients with ARDS [11].

It has been suggested that the addition of biomarkers to the clinical definition of ARDS could reduce ARDS heterogeneity by the identification of subgroups [12–15]. A retrospective latent class analysis of large randomized controlled trials identified two ARDS phenotypes largely based on ARDS biomarkers combined with clinical parameters [16, 17]. These phenotypes responded differently to the randomly assigned intervention arms. Prospective studies are required to validate these ARDS phenotypes and their response to interventions. The set of ARDS biomarkers to prospectively define these phenotypes remains to be established.

Numerous biomarkers and their pathophysiological role in ARDS have been described [12, 18]. In an earlier meta-analysis, biomarkers for ARDS development and mortality were examined in univariate analysis [19]. However, pooling of univariate biomarker data may result in overestimation of the actual effect. For this reason, we conducted a systematic review and included all biomarkers that were multivariately associated with ARDS development or mortality. This study provides a synopsis of ARDS biomarkers that could be used for future research in the identification of ARDS phenotypes.

## Methods

This systematic review was prospectively registered in PROSPERO International Prospective Register of Systematic Reviews (PROSPERO identifier CRD42017078957) and performed according to the Transparent Reporting of Systematic Reviews and Meta-analyses (PRISMA) Statement [20]. After the search strategy, two reviewers (PZ, PS, and/or WG) separately performed study eligibility criteria, data extraction, and quality assessment. Any discrepancies were resolved by consensus, and if necessary, a third reviewer was consulted.

We searched for studies that included biomarkers that were associated with ARDS development in critically ill patients at risk for ARDS and mortality in the ARDS population in multivariate analyses adjusted for background characteristics. We did not perform a meta-analysis, because the raw data in all studies was either not transformed or log transformed resulting in varying risk ratios and confidence intervals. In addition, the majority of studies used different biomarker concentration cut-offs, resulting in varying concentration increments for risk ratios. Lastly, the number of days until mortality and variables used in multivariate analysis differed between studies. For these reasons, we limited this study to a systematic review, as the multivariate odds ratios were not comparable and pooling would result in non-informative estimates [21].

### Search strategy

We performed a systematic search in Embase, MEDLINE, Web of Science, Cochrane CENTRAL, and Google Scholar from inception until 30 July 2018 with assistance from the Erasmus MC librarian. The search was later updated to 6 March 2020. A detailed description of the systematic search string is presented in Additional file 1. In addition, the reference lists of included studies and recent systematic reviews were screened to identify additional eligible studies.

### Study eligibility criteria

All retrieved studies were screened on the basis of title and abstract. Studies that did not contain adult patients at risk for ARDS or with ARDS and any biomarker for ARDS were excluded. The following eligibility criteria were used: human research, adult population, studies in which biomarkers were presented as odds ratios (OR) or risk ratios in multivariate analysis with ARDS development or mortality as outcome of interest, peer-reviewed literature only, and English language. Studies comparing ARDS with healthy control subjects, case series (< 10 patients included in the study), and studies presenting gene expression fold change were excluded.

### Data extraction

A standardized form was used for data extraction from all eligible studies. Two clinical endpoints were evaluated in this study: development of ARDS in the at-risk population (patients that did develop ARDS versus critically ill patients that did not) and mortality in the ARDS population (survivors versus non-survivors). The following data were extracted: study design and setting, study population, sample size, the definition of ARDS used in the study, outcome, risk ratio with 95% confidence interval in multivariate analyses, and the variables used in the analyses. In addition, the role of the biomarker in ARDS pathophysiology as reported by the studies was extracted and divided into the following categories: increased endothelial permeability, alveolar epithelial injury, oxidative injury, inflammation, pro-fibrotic, myocardial strain, coagulation, and others. Subsequently, the relative frequency distribution of biomarker roles in ARDS pathophysiology was depicted in a bar chart.

### Quality assessment

Methodological quality of the included studies was assessed with the Newcastle-Ottawa Scale (NOS) for assessing the quality of nonrandomized studies in systematic reviews and meta-analyses [22]. Items regarding patient selection, comparability, and outcome were assessed using a descriptive approach, and a risk-of-bias score, varying between 0 (high risk) and 9 (low risk), was assigned to each study.

## Results

### Literature search and study selection

**Fig. 1:**
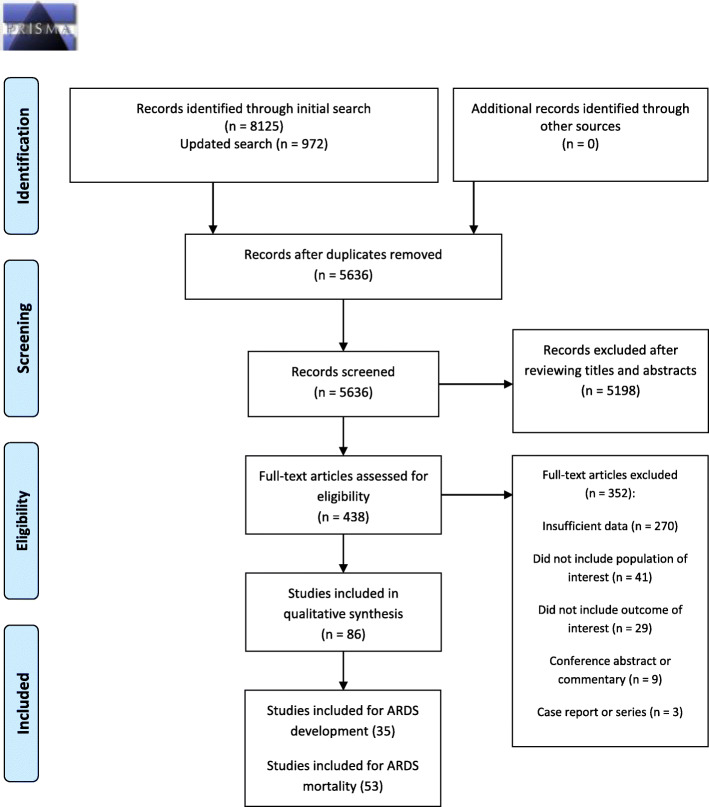
PRISMA flow diagram for a systematic search

A total of 8125 articles were identified by the initial search and 972 by the updated search (Fig. 1). After removal of duplicates and reviewing titles and abstracts, we selected 438 articles for full-text review. A total of 86 studies was eligible for data extraction: 35 for ARDS development and 53 for ARDS mortality.

### Study characteristics and quality assessment

The study characteristics of the 35 studies for ARDS development are presented in Table 1. A total of 10,667 critically ill patients was at risk for ARDS, of whom 2419 (24.6%) patients developed ARDS. The majority of studies used the Berlin definition of ARDS (21/35), followed by the AECC criteria of ARDS (13/35). The included biomarkers were measured in plasma, cerebrospinal fluid, and bronchoalveolar lavage fluid. In all studies, the first sample was taken within 72 h following ICU admission.

**Table 1:** Study characteristics for ARDS development

Study	Study design	Study population	ARDS definition	Outcome	Total (***n***)	ARDS (***n***)	Age	Gender, male ***n*** (%)	Variables in multivariate analysis	Sample moment
Agrawal 2013 [23]	Prospective cohort	Critically ill	AECC	ALI	167	19	69 ± 16	8 (42.1%)	APACHE II score, sepsis	Within 24 h following admission
Ahasic 2012 [24]	Case-control	Critically ill	AECC	ARDS	531	175	60.7 ± 17.6	102 (58.2%)	Age, gender, APACHE III score, BMI, ARDS risk factor	Within 48 h following admission
Aisiku 2016 [25]	RCT (TBI trial)	Critically ill neurotrauma	Berlin	ARDS	200	52	29.0 (19.5 IQR)	50 (96.2%)	Gender, injury severity scale, Glasgow coma scale	Within 24 h following injury
Amat 2000 [26]	Case-control	Critically ill	AECC	ARDS	35	21	54 ± 16	15 (71.4%)	Not specified	At ICU admission
Bai 2017 [27]	Prospective cohort	Critically ill neurotrauma	Berlin	ARDS	50	21	48 (39–57 IQR)	10 (46.7%)	Age, gender, BMI, injury score, blood transfusion, mechanical ventilation, Marshall CT score, Glasgow coma scale	At admission
Bai 2017 [27]	Prospective cohort	Critically ill trauma	Berlin	ARDS	42	16	44 (35–56 IQR)	10 (62.5%)	Age, gender, BMI, injury score, blood transfusion, mechanical ventilation, Marshall CT score, Glasgow coma scale	At admission
Bai 2018 [28]	Prospective cohort	Stroke patients	Berlin	ARDS	384	60	64 (43–72 IQR)	22 (36.7%)	Age, gender, BMI, onset to treatment time, medical history	Within 6 h following stroke
Chen 2019 [29]	Case-control	Critically ill sepsis	Berlin	ARDS	115	57	56.3 ± 10.1	40 (70.2%)	Age, gender, BMI, smoking history, COPD, cardiomyopathy, APACHE II score, SOFA score	Within 24 h following ARDS onset or ICU admission
Du 2016 [30]	Prospective cohort	Cardiac surgery patients	AECC	ALI	70	18	57.7 ± 11.6	12 (66.7%)	Age, medical history, BMI, systolic blood pressure	Within 1 h following surgery
Faust 2020 [31]	Prospective cohort	Critically ill trauma	Berlin	ARDS	224	41	44 (30–60 IQR)	37 (90.2%)	Injury severity score, blunt mechanism, pre-ICU shock	At ED
Faust 2020 [31]	Prospective cohort	Critically ill sepsis	Berlin	ARDS	120	45	62 (52–67 IQR)	15 (33.3%)	Lung source of sepsis, shock, age	At ED
Fremont 2010 [32]	Case-control	Critically ill	AECC	ALI/ARDS	192	107	39 (26–53 IQR)	71 (66.4%)	Not specified	Within 72 h following ICU admission
Gaudet 2018 [33]	Prospective cohort	Critically ill patients	Berlin	ARDS	72	11	56 (51–63 IQR)	8 (72.7%)	Not specified	At inclusion
Hendrickson 2018 [34]	Retrospective cohort	Severe traumatic brain injury	Berlin	ARDS	182	50	44 ± 20	42 (84.0%)	Age, acute injury scale, Glasgow coma scale, vasopressor use	Within 10 min following ED arrival
Huang 2019 [35]	Prospective cohort	Critically ill sepsis	Berlin	ARDS	152	41	63.2 ± 11.0	32 (78.0%)	Age, gender, BMI, smoking history, COPD, cardiomyopathy, APACHE II score, SOFA score	Within 24 h following ICU admission
Huang 2019 [36]	Prospective cohort	Critically ill pancreatitis	Berlin	ARDS	1933	143	49 (42–60 IQR)	87 (60.8%)	Age, gender, aetiology of ARDS, APACHE II score	At admission
Jabaudon 2018 [37]	Prospective cohort	Critically ill	Berlin	ARDS	464	59	62 ± 16	46 (78.0%)	SAPS II, sepsis, shock, pneumonia	Within 6 h following ICU admission
Jensen 2016 [38]	RCT (PASS)	Critically ill	Berlin	ARDS	405	31	NR	NR	Age, gender, APACHE II score, sepsis, eGFR	Within 24 h following admission
Jensen 2016 [38]	RCT (PASS)	Critically ill	Berlin	ARDS	353*	31	NR	NR	Age, gender, APACHE II score, sepsis, eGFR	Within 24 h following admission
Jones 2020 [39]	Prospective cohort	Critically ill sepsis	Berlin	ARDS	672	261	60 (51–69 IQR)	154 (59.0%)	Pulmonary source, APACHE III score	At admission
Jones 2020 [39]	Prospective cohort	Critically ill sepsis	Berlin	ARDS	843	NR	NR	NR	Pulmonary source, APACHE III score	Within 48 h following admission
Komiya 2011 [40]	Cross sectional	Acute respiratory failure	AECC	ALI/ARDS	124	53	78 (69–85 IQR)	34 (64.2%)	Age, systolic blood pressure, VEF, chest X-ray pleural effusion	Within 2 h following emergency department arrival
Lee 2011 [41]	Prospective cohort	Critically ill	AECC	ALI/ARDS	113	50	57.6 ± 19.1	24 (48.0%)	Sepsis, BMI	Within 24 h following ICU admission
Lin 2017 [42]	Retrospective cohort	Critically ill	Berlin	ARDS	212	83	54.3 ± 20.3	53 (63.9%)	CRP, albumin, serum creatinine, APACHE II score	Within 2 h following ICU admission
Liu 2017 [43]	Prospective cohort	Critically ill	AECC	ALI/ARDS	134	19	69 ± 18	10 (52.6%)	APACHE II, sepsis severity	On arrival at ED
Luo 2017 [44]	Retrospective cohort	Severe pneumonia	AECC	ALI/ARDS	157	43	56 ± 19	25 (58.1%)	Lung injury score, SOFA score, PaO_2_/FiO_2_, blood urea	Day 1 following admission
Meyer 2017 [45]	Prospective cohort	Critically ill trauma	Berlin	ARDS	198	100	60 ± 14	62 (62.0%)	APACHE III score, age, gender, ethnicity, pulmonary infection	On arrival at ED or ICU
Mikkelsen 2012 [46]	Case-control	Critically ill	AECC	ALI/ARDS	48	24	38 ± 20	22 (91.7%)	APACHE III score	In ED
Osaka 2011 [47]	Prospective cohort	Pneumonia	AECC	ALI/ARDS	27	6	75 (51–92 range)	4 (66.7%)	Not specified	3 to 5 days following admission
Palakshappa 2016 [48]	Prospective cohort	Critically ill	Berlin	ARDS	163	73	58 (52–68 IQR)	42 (57.5%)	APACHE III score, diabetes, BMI, pulmonary sepsis	At ICU admission
Reilly 2018 [49]	Prospective cohort	Critically ill sepsis	Berlin	ARDS	703	289	60 (51–69 IQR)	170 (58.8%)	Pulmonary source, APACHE III score	Within 24 h of ICU admission
Shashaty 2019 [50]	Prospective cohort	Critically ill sepsis	Berlin	ARDS	120	44	61 (50–68 IQR)	NR	Age, transfusion, pulmonary source, shock	At ED
Shashaty 2019 [50]	Prospective cohort	Critically ill trauma	Berlin	ARDS	180	37	41 (25–62 IQR)	NR	Injury severity score, blunt mechanism, transfusion	At presentation
Shaver 2017 [51]	Prospective cohort	Critically ill	AECC	ARDS	280	90	54 (44–64 IQR)	54 (60.0%)	Age, APACHE II, sepsis	Day of inclusion
Suzuki 2017 [52]	Retrospective cohort	Suspected drug-induced lung injury	New bilateral lung infiltration	ALI/ARDS	68	39	72 (65-81IQR)	25 (64.1%)	Gender, age, smoking history, biomarkers	As soon as possible after DLI suspicion
Wang 2019 [53]	Prospective cohort	Critically ill sepsis	Berlin	ARDS	109	32	58 ± 10.7	NR	Age, gender, BMI, smoking history, COPD, cardiomyopathy, APACHE II score, SOFA score	Within 24 h following admission
Ware 2017 [54]	Prospective cohort	Critically ill trauma patients	Berlin	ARDS	393	78	42 (26–55)	56 (71.8%)	Not specified	Within 24 h following inclusion
Xu 2018 [55]	Prospective cohort	Critically ill	Berlin	ARDS	158	45	60.0 ± 17.1	35 (77.8%)	APACHE II score, Lung injury prediction score, biomarkers, sepsis	Within 24 h of ICU admission
Yeh 2017 [56]	Prospective cohort	Critically ill	AECC	ALI/ARDS	129	18	65 ± 18	10 (55.6%)	APACHE II score	On arrival at the ED
Ying 2019 [57]	Prospective cohort	Critically ill pneumonia	Berlin	ARDS	145	37	61.3 ± 10.4	23 (62.2%)	Age, SOFA score, lung injury score, heart rate	At admission
				**Total**^†^	10,667	2419				
						24.6%				

*Validating cohort

^†^Some studies included patients from the same cohort

*Abbreviations*: *AECC* American European Consensus Conference definition of ARDS, *ALI* acute lung injury, *APACHE* acute physiology and chronic health evaluation, *ARDS* acute respiratory distress syndrome, *BMI* body mass index, *COPD* chronic obstructive pulmonary disease, *CRP C*-reactive protein, *DLI* drug-induced lung injury, *ED* emergency department, *eGFR* estimated glomerular filtration rate, *ICU* intensive care unit, *LVEF* left ventricular ejection fraction, *SAPS* simplified acute physiology score, *SOFA* sequential organ failure assessment

The study characteristics of the 53 studies for ARDS mortality are presented in Table 2. A total of 15,344 patients with ARDS were included with an observed mortality rate of 36.0%. The AECC definition of ARDS was used in the majority of included studies (39/53). The included biomarkers were measured in plasma, bronchoalveolar lavage fluid, and urine. All samples were taken within 72 h following the development of ARDS.

**Table 2:** Study characteristics for ARDS mortality

Study	Study design	Setting	ARDS definition	Outcome	Total (***n***)	Non-survivors (***n***)	Age	Gender, male ***n*** (%)	Variables in multivariate analysis	Sample moment
Adamzik 2013 [58]	Prospective cohort	Single centre	AECC	30 days	47	17	44 ± 13	32 (68. 1%)	SAPS II score, gender, lung injury score, ECMO, CVVHD, BMI, CRP, procalcitonin	Within 24 h following ICU admission
Ahasic 2012 [24]	Prospective cohort	Multicentre	AECC	60 days	175	78	60.7 ± 17.6	102 (58.3%)	Gender, BMI, cirrhosis, Diabetes, need for red cell transfusion, sepsis, septic shock, trauma	Within 48 h following ICU admission
Amat 2000 [26]	Prospective cohort	Two centre	AECC ARDS	1 month after ICU discharge	21	11	54 ± 16	15 (71.4%)	Not specified	Day 0 ICU
Bajwa 2008 [59]	Prospective cohort	Single centre	AECC	60 day	177	70	68.3 ± 15.3	99 (55.9%)	APACHE III score	Within 48 h following ARDS onset
Bajwa 2009 [60]	Prospective cohort	Single centre	AECC	60 days	177	70	62.5 (IQR 29.0)	100 (56.5%)	APACHE III score	Within 48 h following ARDS onset
Bajwa 2013 [61]	RCT (FACTT)	Multicentre	AECC	60 days	826	NR	48 (38–59 IQR)	442 (53.5%)	APACHE III score	Days 0 and 3
Calfee 2008 [62]	RCT (ARMA)	Multicentre	AECC	180 days	676	NR	51 ± 17	282 (41.7%)	Age, gender, APACHE III score, sepsis, or trauma	Day 0
Calfee 2009 [63]	RCT (ARMA)	Multicentre	AECC	Hospital	778	272	51 ± 17	459 (59.0%)	Age, PaO_2_/FiO_2_, APACHE III score, sepsis or trauma	Day 0
Calfee 2011 [64]	RCT (ARMA)	Multicentre	AECC	90 days	547	186	50 ± 16	227 (41.5%)	APACHE III score, tidal volume	Day 0
Calfee 2012 [65]	RCT (FACTT)	Multicentre	AECC	90 days	931	261	50 ± 16	498 (53.5%)	Age, APACHE III score, fluid management strategy	Day 0
Calfee 2015 [66]	Prospective cohort	Single centre	AECC	Hospital	100	31	58 ± 11	52 (52.0%)	APACHE III score	Day 2 following ICU admission
Calfee 2015 [66]	RCT (FACTT)	Multicentre	AECC	90 days	853	259	51 ± 15	444 (52.1%)	APACHE III score	Within 48 h following ARDS onset
Cartin-Ceba 2015 [67]	Prospective cohort	Single centre	AECC	In-hospital	100	36	62.5 (51–75 IQR)	54 (54.0%)	Acute physiology score of APACHE III score, DNR status, McCabe score	Within 24 h following diagnosis
Chen 2009 [68]	Prospective cohort	Single centre	*	28 days	59	26	62 ± 19	35 (59.3%)	APACHE II score, biomarkers	Within 24 h following diagnosis
Clark 1995 [69]	Prospective cohort	Single centre	**	Mortality	117	48	43.4 ± 15.4	75 (64.1%)	Lung injury score, risk factor for ARDS, lavage protein concentration	Day 3 following disease onset
Clark 2013 [70]	RCT (FACTT)	Multicentre	AECC	60 days	400	106	47 (37–57 IQR)	210 (52.5%)	Age, gender, ethnicity, baseline serum creatinine, ARDS risk factor	Day 1 following inclusion
Dolinay 2012 [71]	Prospective cohort	Single centre	AECC	In-hospital	28	17	54 ± 14.5	13 (46.4%)	APACHE II score	Within 48 h following ICU admission
Eisner 2003 [72]	RCT (ARMA)	Multicentre	AECC	180 days	565	195	51 ± 17	332 (58.8%)	Ventilation strategy, APACHE III score, PaO_2_/FiO_2_, creatinine, platelet count	Day 0 following inclusion
Forel 2015 [73]	Prospective cohort	Multicentrer	Berlin < 200 mmHg	ICU	51	NR (for ICU)	60 ± 13	40 (78.4%)	Lung injury score	Day 3
Forel 2018 [74]	Prospective cohort	Single centre	Berlin < 200 mmHg	60 days	62	21	59 ± 15	47 (75.8%)	Gender, SOFA score, LIS score	Day 3 following onset of ARDS
Guervilly 2011 [75]	Prospective cohort	Single centre	AECC	28 days	52	21	58 ± 17	39 (75.0%)	Not specified	Within 24 h following diagnosis
Kim 2019 [76]	Retrospective cohort	Single centre	Berlin	In-hospital	97	63	67.2 (64.3–70.1)	63 (64.3%)	APACHE II score, SOFA score, SAPS II score	Within 48 h following admission
Lee 2019 [77]	Retrospective cohort	Single centre	Berlin	In-hospital	237	154	69 (61–74 IQR)	166 (70.0%)	Age, diabetes mellitus, non-pulmonary source, APACHE II score, SOFA	Within 24 h following intubation
Lesur 2006 [78]	Prospective cohort	Multicentre	AECC	28 days	78	29	63 ± 16	48 (61.5%)	Age, PaCO_2_, APACHE II score	Within 48 h following onset of ARDS
Li 2019 [79]	Retrospective cohort	Single centre	Berlin	28 days	224	70	64 (46–77 IQR)	140 (62.5%)	APACHE II score, age, gender, BMI, smoking status, alcohol abusing status, risk factors, comorbidities	Within 24 h following ICU admission
Lin 2010 [80]	Prospective cohort	Single centre	AECC ARDS	28 days	63	27	75 (57–83 IQR)	38 (60.3%)	Age, lung injury score, SOFA score, APACHE II score, CRP, biomarkers	Within 24 h following ARDS onset
Lin 2012 [81]	Prospective cohort	Single centre	AECC	30 days	87	27	61 (56–70 IQR)	42 (48.3%)	APACHE II, Lung injury score, creatinine, biomarkers	At inclusion
Lin 2013 [82]	Prospective cohort	Single centre	AECC	30 days	78	22	63 (54–68 IQR)	45 (57.7%)	Age, APACHE II score, Lung injury score, PaO_2_/FiO_2_	Within 10 h following diagnosis
Madtes 1998 [83]	Prospective cohort	Single centre	***	In-hospital	74	33	38 (19–68 Range)	50 (67.6%)	Age, PCP III levels, neutrophils, lung injury score	Day 3 following ARDS onset
McClintock 2006 [84]	RCT (ARMA)	Multicentre	AECC	Mortality	579	NR	51 ± 17	333 (57.5%)	Ventilator group assignment	Day 0 following inclusion
McClintock 2007 [85]	RCT (ARMA)	Multicentre	AECC	Mortality	576	NR	52 ± 17	328 (56.9%)	Gender, ventilator group assignment, eGFR, age, APACHE III score, vasopressor use, sepsis	Day 0 following inclusion
McClintock 2008 [86]	Prospective cohort	Two centre	AECC	In-hospital	50	21	55 ± 16	28 (56.0%)	Age, gender, SAPS II	Within 48 h following diagnosis
Menk 2018 [87]	Retrospective cohort	Single centre	Berlin	ICU	404	182	50 (37–61 IQR)	265 (65.6%)	Age, gender, APACHE II score, SOFA, severe ARDS, peak airway pressure, pulmonary compliance	Within 24 h following admission
Metkus 2017 [88]	RCT (ALVEOLI, FACTT)	Multicentre	AECC	60 days	1057	NR	50.4	549 (51.9%)	Age, gender, trial group assignment	Within 24 h following inclusion
Mrozek 2016 [89]	Prospective cohort	Multicentre	AECC	90 days	119	42	57 ± 17	82 (68.9%)	Age, gender, SAPS II score, PaO_2_/FiO_2,_ sepsis	Within 24 h following inclusion
Ong 2010 [90]	Prospective cohort	Two centre	AECC	28-day in-hospital	24	NR	51 ± 21	30 (53.6%)	Age, gender, PaO_2_/FiO_2_, tidal volume, plateau pressure, APACHE II score	At inclusion
Parsons 2005 [91]	RCT (ARMA)	Multicentre	AECC	180 days or discharge	562	196	NR	NR	Ventilation strategy, APACHE III score, PaO_2_/FiO_2_, creatinine, platelet count, vasopressor use	At inclusion
Parsons 2005 [92]	RCT (ARMA)	Multicentre	AECC	In-hospital	781	276	51.6 ± 17.3	319 (40.1%)	Ventilation strategy, APACHE III score, PaO_2_/FiO_2_, creatinine, platelet count, vasopressor use	Day 0
Quesnel 2012 [93]	Prospective cohort	Single centre	AECC	28 days	92	37	67 (49–74 IQR)	61 (66.3%)	Age, SAPS II score, malignancy, SOFA score, BAL characteristics	NR
Rahmel 2018 [94]	Retrospective cohort	Single centre	AECC	30 days	119	37	43.7 ± 13.3	71 (59.7%)	Age, SOFA score	Within 24 h following admission
Reddy 2019 [95]	Prospective cohort	Single centre	Berlin	30 days	39	19	55 (47.5-61.5)	25 (64.1%)	Not specified	Within 24 h of ARDS diagnosis
Rivara 2012 [96]	Prospective cohort	Single centre	AECC	60 days	177	70	71.5 (59–80 IQR)	98 (55.4%)	APACHE III score	Within 48 h following diagnosis
Rogers 2019 [97]	RCT (SAILS)	Multicentre	AECC	60 days	683	NR	56 (43–65)	335 (49.0%)	Age, race, APACHE III score, GFR, randomization, shock	Within 48 h following ARDS diagnosis
Sapru 2015 [98]	RCT (FACTT)	Multicentre	AECC	60 days	449	109	49.8 ± 15.6	242 (53.9%)	Age, gender, APACHE III score, pulmonary sepsis, fluid management strategy	Upon inclusion
Suratt 2009 [99]	RCT (ARMA)	Multicentre	AECC	In-hospital	645	222	51 ± 17	381 (59.1%)	Ventilation strategy, age, gender	Day 0
Tang 2014 [100]	Prospective cohort	Multicentre	Berlin	In-hospital	42	20	72.5 ± 10.8	27 (64.3%)	APACHE II score, PaO_2_/FiO_2_, CRP, WBC, procalcitonin	Within 24 h following diagnosis
Tsangaris 2009 [101]	Prospective cohort	Single centre	AECC	28 days	52	27	66.1 ± 16.9	32 (59.6%)	APACHE II score, age, genotype	Within 48 h following admission
Tsangaris 2017 [102]	Prospective cohort	Single centre	NR	28 days	53	28	64.6 ± 16.8	33 (62.3%)	Lung injury score	Within 48 h following diagnosis
Tsantes 2013 [103]	Prospective cohort	Single centre	AECC	28 days	69	34	64.4 ± 17.9	43 (62.3%)	Age, gender, APACHE II score, SOFA score, pulmonary parameters, serum lactate	Within 48 h following diagnosis
Tseng 2014 [104]	Prospective cohort	Single centre	AECC ARDS	ICU	56	16	70.6 ± 9.2	31 (55.4%)	APACHE II score, SOFA score, SAPS II score	Day 1 following ICU admission
Wang 2017 [105]	Prospective cohort	Multicentre	Berlin	60 days	167	62	76.5 (19–95 range)	112 (67.1%)	Age, gender, APACHE II score	Day 1 following diagnosis
Wang 2018 [106]	Retrospective cohort	Single centre	AECC	Mortality	247	146	62 (48–73 IQR)	162 (65.6%)	Age, cirrhosis, creatinine, PaO_2_/FiO_2_	Within 24 h following diagnosis
Ware 2004 [107]	RCT (ARMA)	Multicentre	AECC	In-hospital	559	193	51 ± 17	332 (59.4%)	Ventilator strategy, APACHE III score, PaO_2_/FiO_2_, creatinine, platelet count	Day 0 of inclusion
Xu 2017 [108]	Retrospective cohort	Single centre	Berlin	28 days	63	27	54 (42–67 IQR)	37 (58.7%)	APACHE II score, PaO_2_/FiO_2_, procalcitonin	Within 48 following admission
				**Total**^†^	15,344	3914				
						36.0%				

*Respiratory failure requiring positive pressure ventilation, PF ratio < 200 mmHg, bilateral pulmonary infiltration on chest X-ray, no clinical evidence of left atrial hypertension

**PF ratio < 150 mmHg, PF < 200 mmHg with 5 PEEP, diffuse parenchymal infiltrates, pulmonary artery wedge pressure < 18 mmHg, no clinical evidence of congestive heart failure

***PF ratio < 150 mmHg, PF ratio < 200 mmHg with 5 cmH2O PEEP, diffuse parenchymal infiltrates, pulmonary artery wedge pressure < 18 mmHg, or no clinical evidence of congestive heart failure

^†^Some studies included patients from the same cohort

*Abbreviations*: *AECC* American European Consensus Conference definition of ARDS, *APACHE* acute physiology and chronic health evaluation, *ARDS* acute respiratory distress syndrome, *BAL* bronchoalveolar lavage, *BMI* body mass index, *CRP* C-reactive protein, *CVVHD* continuous veno-venous haemodialysis, *DNR* do not resuscitate, *ECMO* extra corporeal membrane oxygenation, *eGFR* estimated glomerular filtration rate, *FiO*_*2*_ fraction of inspired oxygen, *ICU* intensive care unit, *PCP* procollagen, *No*. number, *SAPS* simplified acute physiology score, *SOFA* sequential organ failure assessment, *WBC* white blood cell count

The median quality of the included publications according to the NOS was 7 (range 4–9) for ARDS development and 8 (range 5–9) for ARDS mortality (Additional file 2).

### Biomarkers associated with ARDS development in the at-risk population

**Fig. 2:**
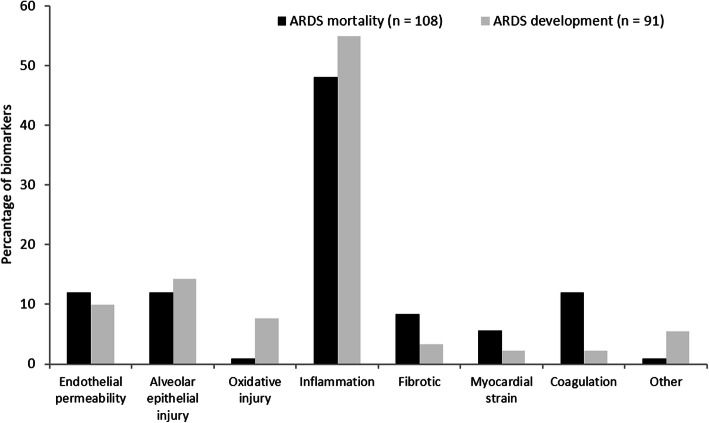
Biomarker role in ARDS pathophysiology

A total of 37 biomarkers in plasma, 7 in cerebrospinal fluid, and 1 in bronchoalveolar lavage fluid were assessed in multivariate analyses (Table 3). Five studies examined angiopoeitin-2 (Ang-2) and seven studies examined receptor for advanced glycation end products (RAGE). In all studies, high plasma levels of Ang-2 and RAGE were significantly associated with an increased risk of ARDS development in the at-risk population. Similar results were seen for surfactant protein D (SpD) in plasma in all three studies that assessed SpD. In contrast, biomarkers for inflammation as C-reactive protein (CRP), procalcitonin, interleukin-6, and interleukin-8 were not clearly associated with ARDS development. The majority of biomarkers in plasma are surrogates for inflammation in ARDS pathophysiology (Fig. 2).

**Table 3:** Risk ratios for ARDS development in the at-risk population

	Reference	Biomarker role in ARDS	Sample size	Risk ratio (95% CI)	Cut-off	Comment
**Biomarkers in plasma**						
Adiponectin	Palakshappa 2016 [48]	Anti-inflammatory	163	1.12 (1.01–1.25)	Per 5 mcg/mL	
Angiopoietin-2	Agrawal 2013 [23]	Increased endothelial permeability	167	1.8 (1.0–3.4)	Per log10	
Angiopoietin-2	Fremont 2010 [32]	Increased endothelial permeability	192	2.20 (1.19–4.05)	Highest vs lowest quartile	
Angiopoietin-2	Reilly 2018 [49]	Increased endothelial permeability	703	1.49 (1.20–1.77)	Per log increase	
Angiopoietin-2	Ware 2017 [54]	Increased endothelial permeability	393	1.890 (1.322–2.702)	1st vs 4th quartile	
Angiopoietin-2	Xu 2018 [55]	Increased endothelial permeability	158	1.258 (1.137–1.392)		
Advanced oxidant protein products	Du 2016 [30]	Oxidative injury	70	1.164 (1.068–1.269)		
Brain natriuretic peptide	Fremont 2010 [32]	Myocardial strain	192	0.45 (0.26–0.77)	Highest vs lowest quartile	
Brain natriuretic peptide	Komiya 2011 [40]	Myocardial strain	124	14.425 (4.382–47.483)	> 500 pg/mL	Outcome is CPE
Club cell secretory protein	Jensen 2016 [38]	Alveolar epithelial injury	405	2.6 (0.7–9.7)	≥ 42.8 ng/mL	Learning cohort
Club cell secretory protein	Jensen 2016 [38]	Alveolar epithelial injury	353	0.96 (0.20–4.5)	≥ 42.8 ng/mL	Validating cohort
Club cell secretory protein	Lin 2017 [42]	Alveolar epithelial injury	212	1.096 (1.085–1.162)		
C-reactive protein (CRP)	Bai 2018 [28]	Inflammation	384	1.314 (0.620–1.603)		
C-reactive protein (CRP)	Chen 2019 [29]	Inflammation	115	0.994 (0.978–1.010)		
C-reactive protein (CRP)	Huang 2019 [35]	Inflammation	152	1.287 (0.295–5.606)	≥ 90.3 mg/L	
C-reactive protein (CRP)	Huang 2019 [36]	Inflammation	1933	1.008 (1.007–1.010)		
C-reactive protein (CRP)	Komiya 2011 [40]	Inflammation	124	0.106 (0.035–0.323)	> 50 mg/L	Outcome is CPE
C-reactive protein (CRP)	Lin 2017 [42]	Inflammation	212	1.007 (1.001–1.014)		
C-reactive protein (CRP)	Osaka 2011 [47]	Inflammation	27	1.029 (0.829–1.293)	Per 1 mg/dL increase	
C-reactive protein (CRP)	Wang 2019 [53]	Inflammation	109	1.000 (0.992–1.008)		
C-reactive protein (CRP)	Ying 2019 [57]	Inflammation	145	1.22 (0.95–1.68)		
Free 2-chlorofatty acid	Meyer 2017 [45]	Oxidative injury	198	1.62 (1.25–2.09)	Per log10	
Total 2-chlorofatty acid	Meyer 2017 [45]	Oxidative injury	198	1.82 (1.32–2.52)	Per log10	
Free 2-chlorostearic acid	Meyer 2017 [45]	Oxidative injury	198	1.82 (1.41–2.37)	Per log10	
Total 2-chlorostearic acid	Meyer 2017 [45]	Oxidative injury	198	1.78 (1.31–2.43)	Per log10	
Endocan	Gaudet 2018 [33]	Leukocyte adhesion inhibition	72	0.001 (0–0.215)	> 5.36 ng/mL	
Endocan	Mikkelsen 2012 [46]	Leukocyte adhesion inhibition	48	0.69 (0.49–0.97)	1 unit increase	
Endocan	Ying 2019 [57]	Leukocyte adhesion modulation	145	1.57 (1.14–2.25)		
Fibrinogen	Luo 2017 [44]	Coagulation	157	1.893 (1.141–3.142)		
Glutamate	Bai 2017 [27]	Non-essential amino acid, neurotransmitter	50	2.229 (1.082–2.634)		
Glutamate	Bai 2017 [27]	Non-essential amino acid, neurotransmitter	42	0.996 (0.965–1.028)		
Glutamate	Bai 2018 [28]	Non-essential amino acid	384	3.022 (2.001–4.043)		
Growth arrest-specific gene 6	Yeh 2017 [56]	Endothelial activation	129	1.6 (1.3–2.6)		
Insulin-like growth factor 1	Ahasic 2012 [24]	Pro-fibrotic	531	0.58 (0.42–0.79)	Per log10	
IGF binding protein 3	Ahasic 2012 [24]	Pro-fibrotic	531	0.57 (0.40–0.81)	Per log10	
Interleukin-1 beta	Aisiku 2016 [25]	Pro-inflammatory	194	0.98 (0.73–1.32)		
Interleukin-1 beta	Chen 2019 [29]	Pro-inflammatory	115	1.001 (0.945–1.061)		
Interleukin-1 beta	Huang 2019 [35]	Pro-inflammatory	152	0.666 (0.152–2.910)	≥ 11.3 pg/mL	
Interleukin-1 beta	Wang 2019 [53]	Pro-inflammatory	109	1.021 (0.982–1.063)		
Interleukin-6	Aisiku 2016 [25]	Pro-inflammatory	195	1.24 (1.05–1.49)		
Interleukin-6	Bai 2018 [28]	Pro-inflammatory	384	1.194 (0.806–1.364)		
Interleukin-6	Chen 2019 [29]	Pro-inflammatory	115	0.998 (0.993–1.003)		
Interleukin-6	Huang 2019 [35]	Pro-inflammatory	152	0.512 (0.156–1.678)	≥ 63.7 pg/mL	
Interleukin-6	Yeh 2017 [56]	Pro-inflammatory	129	1.4 (0.98–1.7)		
Interleukin-8	Agrawal 2013 [23]	Pro-inflammatory	167	1.3 (0.97–1.8)	Per log10	
Interleukin-8	Aisiku 2016 [25]	Pro-inflammatory	194	1.26 (1.04–1.53)		
Interleukin-8	Chen 2019 [29]	Pro-inflammatory	115	1.000 (0.996–1.003)		
Interleukin-8	Fremont 2010 [32]	Pro-inflammatory	192	1.81 (1.03–3.17)	Highest vs lowest quartile	
Interleukin-8	Liu 2017 [43]	Pro-inflammatory	134	1.4 (0.98–1.7)	Per log10	
Interleukin-8	Yeh 2017 [56]	Pro-inflammatory	129	1.4 (0.92–1.7)		
Interleukin-10	Aisiku 2016 [25]	Anti-inflammatory	195	1.66 (1.22–2.26)		
Interleukin-10	Chen 2019 [29]	Anti-inflammatory	115	1.003 (0.998–1.018)		
Interleukin-10	Fremont 2010 [32]	Anti-inflammatory	192	2.02 (0.96–4.25)	Highest vs lowest quartile	
Interleukin-12p70	Aisiku 2016 [25]	Pro-inflammatory	194	1.18 (0.82–1.69)		
Interleukin-17	Chen 2019 [29]	Pro-inflammatory	115	1.003 (1.000–1.007)		Not significant
Interleukin-17	Huang 2019 [35]	Pro-inflammatory	152	0.644 (0.173–2.405)	≥ 144.55 pg/mL	
Interleukin-17	Wang 2019 [53]	Pro-inflammatory	109	1.001 (0.997–1.004)		
Leukotriene B4	Amat 2000 [26]	Pro-inflammatory	35	14.3 (2.3–88.8)	> 14 pmol/mL	
Microparticles	Shaver 2017 [51]	Coagulation	280	0.693 (0.490–0.980)	Per 10 μM	
Mitochondrial DNA	Faust 2020 [31]	Damage-associated molecular pattern	224	1.58 (1.14–2.19)		48 h plasma
Mitochondrial DNA	Faust 2020 [31]	Damage-associated molecular pattern	120	1.52 (1.12–2.06)	Per log copies per microlitre	48 h plasma
Myeloperoxidase	Meyer 2017 [45]	Pro-inflammatory	198	1.28 (0.89–1.84)	Per log10	
Nitric oxide	Aisiku 2016 [25]	Oxidative injury	193	1.60 (0.98–2.90)		
Parkinson disease 7	Liu 2017 [43]	Anti-oxidative injury	134	1.8 (1.1–3.5)	Per log10	
Pre B cell colony enhancing factor	Lee 2011 [41]	Pro-inflammatory	113	0.78 (0.43–1.41)	Per 10 fold increase	
Procalcitonin	Bai 2018 [28]	Inflammation	384	1.156 (0.844–1.133)		
Procalcitonin	Chen 2019 [29]	Inflammation	115	1.020 (0.966–1.077)		
Procalcitonin	Huang 2019 [35]	Inflammation	152	2.506 (0.705–8.913)	≥ 13.2 ng/mL	
Procalcitonin	Huang 2019 [36]	Inflammation	1933	1.008 (1.000–1.016)		Not significant
Procalcitonin	Wang 2019 [53]	Inflammation	109	1.019 (0.981–1.058)		
Procollagen III	Fremont 2010 [32]	Pro-fibrotic	192	2.90 (1.61–5.23)	Highest vs lowest quartile	
Receptor for advanced glycation end products	Fremont 2010 [32]	Alveolar epithelial injury	192	3.33 (1.85–5.99)	Highest vs lowest quartile	
Receptor for advanced glycation end products	Jabaudon 2018 [37]	Alveolar epithelial injury	464	2.25 (1.60–3.16)	Per log10	Baseline
Receptor for advanced glycation end products	Jabaudon 2018 [37]	Alveolar epithelial injury	464	4.33 (2.85–6.56)	Per log10	Day 1
Receptor for advanced glycation end products	Jones 2020 [39]	Alveolar epithelial injury	672	1.73 (1.35–2.21)		European ancestry
Receptor for advanced glycation end products	Jones 2020 [39]	Alveolar epithelial injury	672	2.05 (1.50–2.83)		African ancestry
Receptor for advanced glycation end products	Jones 2020 [39]	Alveolar epithelial injury	843	2.56 (2.14–3.06)		European ancestry
Receptor for advanced glycation end products	Ware 2017 [54]	Alveolar epithelial injury	393	2.382 (1.638–3.464)	1st vs 4th quartile	
Receptor interacting protein kinase-3	Shashaty 2019 [50]	Increased endothelial permeability	120	1.30 (1.03–1.63)	Per 0.5 SD	
Receptor interacting protein kinase-3	Shashaty 2019 [50]	Increased endothelial permeability	180	1.83 (1.35–2.48)	Per 0.5 SD	
Soluble endothelial selectin	Osaka 2011 [47]	Pro-inflammatory	27	1.099 (1.012–1.260)	Per 1 ng/mL increase	
Soluble urokinase plasminogen activator receptor	Chen 2019 [29]	Pro-inflammatory	115	1.131 (1.002–1.277)		
Surfactant protein D	Jensen 2016 [38]	Alveolar epithelial injury	405	3.4 (1.0–11.4)	≥ 525.6 ng/mL	Learning cohort
Surfactant protein D	Jensen 2016 [38]	Alveolar epithelial injury	353	8.4 (2.0–35.4)	≥ 525.6 ng/mL	Validating cohort
Surfactant protein D	Suzuki 2017 [52]	Alveolar epithelial injury	68	5.31 (1.40–20.15)	Per log10	
Tissue inhibitor of matrix metalloproteinase 3	Hendrickson 2018 [34]	Decreases endothelial permeability	182	1.4 (1.0–2.0)	1 SD increase	
Tumour necrosis factor alpha	Aisiku 2016 [25]	Pro-inflammatory	195	1.03 (0.71–1.51)		
Tumour necrosis factor alpha	Chen 2019 [29]	Pro-inflammatory	115	1.002 (0.996–1.009)		
Tumour necrosis factor alpha	Fremont 2010 [32]	Pro-inflammatory	192	0.51 (0.27–0.98)	Highest vs lowest quartile	
Tumour necrosis factor alpha	Huang 2019 [35]	Pro-inflammatory	152	3.999 (0.921–17.375)	≥ 173.0 pg/mL	
Tumour necrosis factor alpha	Wang 2019 [53]	Pro-inflammatory	109	1.000 (0.995–1.005)		
**Biomarkers in CSF**						
Interleukin-1 beta	Aisiku 2016 [25]	Pro-inflammatory	174	1.11 (0.80–1.54)		
Interleukin-6	Aisiku 2016 [25]	Pro-inflammatory	174	1.06 (0.95–1.19)		
Interleukin-8	Aisiku 2016 [25]	Pro-inflammatory	173	1.01 (0.92–1.12)		
Interleukin-10	Aisiku 2016 [25]	Anti-inflammatory	174	1.33 (1.00–1.76)		
Interleukin-12p70	Aisiku 2016 [25]	Pro-inflammatory	173	1.52 (1.04–2.21)		
Nitric oxide	Aisiku 2016 [25]	Oxidative injury	172	1.66 (0.70–3.97)		
Tumour necrosis factor alpha	Aisiku 2016 [25]	Pro-inflammatory	174	1.43 (0.97–2.14)		
**Biomarkers in BALF**						
Soluble trombomodulin	Suzuki 2017 [52]	Endothelial injury	68	7.48 (1.60–34.98)		

*Abbreviations*: *CPE* cardiopulmonary effusion, *CSF* cerebrospinal fluid, *BALF* bronchoalveolar lavage fluid, *SD* standard deviation

### Biomarkers associated with mortality in the ARDS population

A total of 49 biomarkers in plasma, 8 in bronchoalveolar lavage fluid, and 3 in urine were included in this study (Table 4). Ang-2, CRP, interleukin-8 (IL-8), RAGE, SpD, and Von Willebrand factor (VWF) in plasma were assessed in four or more studies. However, none of these biomarkers was associated with ARDS mortality in all four studies. Similarly to biomarkers in ARDS development, the majority of biomarkers for ARDS mortality in plasma had a pathophysiological role in inflammation (Fig. 2). The majority of biomarkers measured in bronchoalveolar lavage fluid had a pro-fibrotic role in ARDS pathophysiology.

**Table 4:** Risk ratios for ARDS mortality in the ARDS population

	Reference	Biomarker role in ARDS	Sample size	Risk ratio (95% CI)	Cut-off	Comment
**Biomarkers in plasma**						
Activin-A	Kim 2019 [76]	Pro-fibrotic	97	2.64 (1.04–6.70)		
Angiopoietin-1/angiopoietin-2 ratio	Ong 2010 [90]	Modulates endothelial permeability	24	5.52 (1.22–24.9)		
Angiopoietin-2	Calfee 2012 [65]	Increased endothelial permeability	931	0.92 (0.73–1.16)	Per log10	Infection-related ALI
Angiopoietin-2	Calfee 2012 [65]	Increased endothelial permeability	931	1.94 (1.15–3.25)	Per log10	Noninfection-related ALI
Angiopoietin-2	Calfee 2015 [66]	Increased endothelial permeability	100	2.54 (1.38–4.68)	Per log10	Single centre
Angiopoietin-2	Calfee 2015 [66]	Increased endothelial permeability	853	1.43 (1.19–1.73)	per log10	Multicentre
Angiotensin 1–9	Reddy 2019 [95]	Pro-fibrotic	39	2.24 (1.15–4.39)	Concentration doubled (in Ln)	
Angiotensin 1–10	Reddy 2019 [95]	Pro-fibrotic	39	0.36 (0.18–0.72)	Concentration doubled (in Ln)	
Angiotensin converting enzyme	Tsantes 2013 [103]	Endothelial permeability, pro-fibrotic	69	1.06 (1.02–1.10)	Per 1 unit increase	28-day mortality
Angiotensin converting enzyme	Tsantes 2013 [103]	Endothelial permeability, pro-fibrotic	69	1.04 (1.01–1.07)	Per 1 unit increase	90-day mortality
NT-pro brain natriuretic peptide	Bajwa 2008 [59]	Myocardial strain	177	2.36 (1.11–4.99)	≥ 6813 ng/L	
NT-pro brain natriuretic peptide	Lin 2012 [81]	Myocardial strain	87	2.18 (1.54–4.46)	Per unit	
Club cell secretory protein	Cartin-Ceba 2015 [67]	Alveolar epithelial injury	100	1.09 (0.60–2.02)	Per log10	
Club cell secretory protein	Lesur 2006 [78]	Alveolar epithelial injury	78	1.37 (1.25–1.83)	Increments of 0.5	
Copeptin	Lin 2012 [81]	Osmo-regulatory	87	4.72 (2.48–7.16)	Per unit	
C-reactive protein (CRP)	Adamzik 2013 [58]	Inflammation	47	1.01 (0.9–1.1)	Per log10	
C-reactive protein (CRP)	Bajwa 2009 [60]	Inflammation	177	0.67 (0.52–0.87)	Per log10	
C-reactive protein (CRP)	Lin 2010 [80]	Inflammation	63	2.316 (0.652–8.226)		
C-reactive protein (CRP)	Tseng 2014 [104]	Inflammation	56	1.265 (0.798–2.005)		Day 3
D-dimer	Tseng 2014 [104]	Coagulation	56	1.211 (0.818–1.793)		
Decoy receptor 3	Chen 2009 [68]	Immunomodulation	59	4.02 (1.20–13.52)	> 1 ng/mL	Validation cohort
Endocan	Tang 2014 [100]	Leukocyte adhesion inhibition	42	1.374 (1.150–1.641)	> 4.96 ng/mL	
Endocan	Tsangaris 2017 [102]	Leukocyte adhesion inhibition	53	3.36 (0.74–15.31)	> 13 ng/mL	
Galectin 3	Xu 2017 [108]	Pro-fibrotic	63	1.002 (0.978–1.029)	Per 1 ng/mL	
Granulocyte colony stimulating factor	Suratt 2009 [99]	Inflammation	645	1.70 (1.06–2.75)	Quartile 4 vs quartile 2	
Growth differentiation factor-15	Clark 2013 [70]	Pro-fibrotic	400	2.86 (1.84–4.54)	Per log10	
Heparin binding protein	Lin 2013 [82]	Inflammation, endothelial permeability	78	1.52 (1.12–2.85)	Per log10	
High mobility group protein B1	Tseng 2014 [104]	Pro-inflammatory	56	1.002 (1.000–1.004)		Day 1
High mobility group protein B1	Tseng 2014 [104]	Pro-inflammatory	56	0.990 (0.968–1.013)		Day 3
Insulin-like growth factor	Ahasic 2012 [24]	Pro-fibrotic	175	0.70 (0.51–0.95)	Per log10	
IGF binding protein 3	Ahasic 2012 [24]	Pro-fibrotic	175	0.69 (0.50–0.94)	Per log10	
Intercellular adhesion molecule-1	Calfee 2009 [63]	Pro-inflammatory	778	1.22 (0.99–1.49)	Per log10	
Intercellular adhesion molecule-1	Calfee 2011 [64]	Pro-inflammatory	547	0.74 (0.59–0.95)	Per natural log	
Intercellular adhesion molecule-1	McClintock 2008 [86]	Pro-inflammatory	50	5.8 (1.1–30.0)	Per natural log	
Interleukin-1 beta	Lin 2010 [80]	Pro-inflammatory	63	1.355 (0.357–5.140)	Per log 10	
Interleukin-6	Calfee 2015 [66]	Pro-inflammatory	100	1.81 (1.34–2.45)	Per log10	Single centre
Interleukin-6	Calfee 2015 [66]	Pro-inflammatory	853	1.24 (1.14–1.35)	Per log10	Multicentre
Interleukin-6	Parsons 2005 [92]	Pro-inflammatory	781	1.18 (0.93–1.49)	Per log10	
Interleukin-8	Amat 2000 [26]	Pro-inflammatory	21	0.09 (0.01–1.35)	> 150 pg/mL	
Interleukin-8	Calfee 2011 [64]	Pro-inflammatory	547	1.36 (1.15–1.62)	Per natural log	
Interleukin-8	Calfee 2015 [66]	Pro-inflammatory	100	1.65 (1.25–2.17)	Per log10	Single centre
Interleukin-8	Calfee 2015 [66]	Pro-inflammatory	853	1.41 (1.27–1.57)	Per log10	Multicentre
Interleukin-8	Cartin-Ceba 2015 [67]	Pro-inflammatory	100	1.08 (0.72–1.61)	Per log10	
Interleukin-8	Lin 2010 [80]	Pro-inflammatory	63	0.935 (0.280–3.114)	Per log 10	
Interleukin-8	McClintock 2008 [86]	Pro-inflammatory	50	2.0 (1.1–4.0)	Per natural log	
Interleukin-8	Parsons 2005 [92]	Pro-inflammatory	780	1.73 (1.28–2.34)	Per log10	
Interleukin-8	Tseng 2014 [104]	Pro-inflammatory	56	1.039 (0.955–1.130)		Day 1
Interleukin-8	Tseng 2014 [104]	Pro-inflammatory	56	1.075 (0.940–1.229)		Day 3
Interleukin-10	Parsons 2005 [92]	Anti-inflammatory	593	1.23 (0.86–1.76)	Per log10	
Interleukin-18	Dolinay 2012 [71]	Pro-inflammatory	28	1.60 (1.17–2.20)	Per 500 pg/mL increase	
Interleukin-18	Rogers 2019 [97]	Pro-inflammatory	683	2.2 (1.5–3.1)	≥ 800 pg/mL	
Leukocyte microparticles	Guervilly 2011 [75]	Immunomodulation	52	5.26 (1.10–24.99)	< 60 elements/μL	
Leukotriene B4	Amat 2000 [26]	Pro-inflammatory	21	22.5 (1.1–460.5)	> 14 pmol/mL	
Neutrophil elastase	Wang 2017 [105]	Pro-inflammatory	167	1.76 (*p* value 0.002)	1 SD change	Day 1
Neutrophil elastase	Wang 2017 [105]	Pro-inflammatory	167	1.58 (*p* value 0.06)	1 SD change	Day 3
Neutrophil elastase	Wang 2017 [105]	Pro-inflammatory	167	1.70 (*p* value 0.001)	1 SD change	Day 7
Neutrophil to lymphocyte ratio	Li 2019 [79]	Pro-inflammatory	224	5.815 (1.824–18.533)	First–fourth quartile	
Neutrophil to lymphocyte ratio	Wang 2018 [106]	Pro-inflammatory	247	1.011 (1.004–1.017)	Per 1% increase	
Neutrophil to lymphocyte ratio	Wang 2018 [106]	Pro-inflammatory	247	1.532 (1.095–2.143)	> 14	
Nucleated red blood cells	Menk 2018 [87]	Erythrocyte progenitor cell, pro-inflammatory	404	3.21 (1.93–5.35)	> 220/μL	
Peptidase inhibitor 3	Wang 2017 [105]	Anti-inflammatory	167	0.50 (*p* value 0.003)	1 SD change	Day 1
Peptidase inhibitor 3	Wang 2017 [105]	Anti-inflammatory	167	0.43 (*p* value 0.001)	1 SD change	Day 3
Peptidase inhibitor 3	Wang 2017 [105]	Anti-inflammatory	167	0.70 (*p* value 0.18)	1 SD change	Day 7
Plasminogen activator inhibitor 1	Cartin-Ceba 2015 [67]	Coagulation	100	0.96 (0.62–1.47)	Per log10	
Plasminogen activator inhibitor 1 (activity)	Tsangaris 2009 [101]	Coagulation	52	1.30 (0.84–1.99)	Per 1 unit increase	
Procalcitonin	Adamzik 2013 [58]	Inflammation	47	1.01 (0.025–1.2)	Per log10	
Procalcitonin	Rahmel 2018 [94]	Inflammation	119	0.999 (0.998–1.001)		
Protein C	McClintock 2008 [86]	Coagulation	50	0.5 (0.2–1.0)	Per natural log	
Protein C	Tsangaris 2017 [102]	Coagulation	53	3.58 (0.73–15.54)	< 41.5 mg/dL	
Receptor for advanced glycation end products	Calfee 2008 [62]	Alveolar epithelial injury	676	1.41 (1.12–1.78)	Per log10	Tidal volume 12 mL/kg
Receptor for advanced glycation end products	Calfee 2008 [62]	Alveolar epithelial injury	676	1.03 (0.81–1.31)	Per log10	Tidal volume 6 mL/kg
Receptor for advanced glycation end products	Calfee 2015 [66]	Alveolar epithelial injury	100	1.98 (1.18–3.33)	Per log10	Single centre
Receptor for advanced glycation end products	Calfee 2015 [66]	Alveolar epithelial injury	853	1.16 (1.003–1.34)	Per log10	Multicentre
Receptor for advanced glycation end products	Cartin-Ceba 2015 [67]	Alveolar epithelial injury	100	0.81 (0.50–1.30)	Per log10	
Receptor for advanced glycation end products	Mrozek 2016 [89]	Alveolar epithelial injury	119	3.1 (1.1–8.9)	–	
Soluble suppression of tumourigenicity-2	Bajwa 2013 [61]	Myocardial strain and inflammation	826	1.47 (0.99–2.20)	≥ 534 ng/mL (day 0)	Day 0
Soluble suppression of tumourigenicity-2	Bajwa 2013 [61]	Myocardial strain and inflammation	826	2.94 (2.00–4.33)	≥ 296 ng/mL (day 3)	Day 3
Soluble triggering receptor expressed on myeloid cells-1	Lin 2010 [80]	Pro-inflammatory	63	6.338 (1.607–24.998)	Per log 10	
Surfactant protein-A	Eisner 2003 [72]	Alveolar epithelial injury	565	0.92 (0.68–1.27)	Per 100 ng/mL increment	
Surfactant protein D	Calfee 2011 [64]	Alveolar epithelial injury	547	1.55 (1.27–1.88)	Per natural log	
Surfactant protein D	Calfee 2015 [66]	Alveolar epithelial injury	100	1.33 (0.82–2.14)	Per log10	Single centre
Surfactant protein D	Calfee 2015 [66]	Alveolar epithelial injury	853	1.09 (0.95–1.24)	Per log10	Multicentre
Surfactant protein D	Eisner 2003 [72]	Alveolar epithelial injury	565	1.21 (1.08–1.35)	Per 100 ng/mL increment	
Thrombin–antithrombin III complex	Cartin-Ceba 2015 [67]	Coagulation	100	1.05 (0.53–2.05)	Per log10	
High sensitivity troponin I	Metkus 2017 [88]	Myocardial injury	1057	0.94 (0.64–1.39)	1st, 5th quintile	
Cardiac troponin T	Rivara 2012 [96]	Myocardial injury	177	1.44 (1.14–1.81)	Per 1 ng/mL increase	
Trombomodulin	Sapru 2015 [98]	Coagulation	449	2.40 (1.52–3.83)	Per log10	Day 0
Trombomodulin	Sapru 2015 [98]	Coagulation	449	2.80 (1.69–4.66)	Per log10	Day 3
Tumour necrosis factor alpha	Lin 2010 [80]	Pro-inflammatory	63	3.691 (0.668–20.998)	Per log 10	
Tumour necrosis factor receptor-1	Calfee 2011 [64]	Pro-inflammatory	547	1.58 (1.20–2.09)	Per natural log	
Tumour necrosis factor receptor-1	Parsons 2005 [91]	Pro-inflammatory	562	5.76 (2.63–12.6)	Per log10	
Tumour necrosis factor receptor-2	Parsons 2005 [91]	Pro-inflammatory	376	2.58 (1.05–6.31)	Per log10	
Uric acid	Lee 2019 [77]	Antioxidant	237	0.549 (0.293–1030)	≥ 3.00 mg/dL	
Von Willebrand factor	Calfee 2011 [64]	Endothelial activation, coagulation	547	1.57 (1.16–2.12)	Per natural log	
Von Willebrand factor	Calfee 2012 [65]	Endothelial activation, coagulation	931	1.51 (1.20–1.90)	Per log10	
Von Willebrand factor	Calfee 2015 [66]	Endothelial activation, coagulation	853	1.83 (1.46–2.30)	Per log10	Multicentre
Von Willebrand factor	Cartin-Ceba 2015 [67]	Endothelial activation, coagulation	100	2.93 (0.90–10.7)	Per log10	
Von Willebrand factor	Ware 2004 [107]	Endothelial activation, coagulation	559	1.6 (1.4–2.1)	Per SD increment	
**Biomarkers in BALF**						
Angiopoietin-2	Tsangaris 2017 [102]	Increased endothelial permeability	53	11.18 (1.06–117.48)	> 705 pg/mL	
Fibrocyte percentage	Quesnel 2012 [93]	Pro-fibrotic	92	6.15 (2.78–13.64)	> 6%	
Plasminogen activator inhibitor 1 (activity)	Tsangaris 2009 [101]	Coagulation	52	0.37 (0.06–2.35)	Per 1 unit increase	
Procollagen III	Clark 1995 [69]	Pro-fibrotic	117	3.6 (1.2–10.7)	≥ 1.75 U/mL	
Procollagen III	Forel 2015 [73]	Pro-fibrotic	51	5.02 (2.06–12.25)	≥ 9 μg/L	
Transforming growth factor alpha	Madtes 1998 [83]	Pro-fibrotic	74	2.3 (0.7–7.0)	> 1.08 pg/mL	
Transforming growth factor beta 1	Forel 2018 [74]	Pro-fibrotic	62	1003 (0.986–1.019)		
T regulatory cell/CD4+ lymphocyte ratio	Adamzik 2013 [58]	Immunomodulation	47	6.5 (1.7–25)	≥ 7.4%	
**Biomarkers in urine**						
Desmosine-to-creatinine ratio	McClintock 2006 [84]	Alveolar epithelial injury (elastin breakdown)	579	1.36 (1.02–1.82)	Per log10	
Nitric oxide	McClintock 2007 [85]	Oxidative injury	576	0.33 (0.20–0.54)	Per log10	
Nitric oxide-to-creatinine ratio	McClintock 2007 [85]	Oxidative injury	576	0.43 (0.28–0.66)	Per log10	

*Abbreviations*: *ALI* acute lung injury, *BALF* bronchoalveolar lavage fluid, *SD* standard deviation

## Discussion

In the current systematic review, we present a synopsis of biomarkers for ARDS development and mortality tested in multivariate analyses. We did not perform a meta-analysis because of severe data heterogeneity between studies. Upon qualitative inspection, we found that high levels of Ang-2 and RAGE were associated with ARDS development in the at-risk population. None of the biomarkers assessed in four or more studies was associated with an increased mortality rate in all studies. The majority of plasma biomarkers for both ARDS development and mortality are surrogates for inflammation in ARDS pathophysiology.

Previously, Terpstra et al. [19] calculated univariate ORs from absolute biomarker concentrations and performed a meta-analysis. They found that 12 biomarkers in plasma were associated with mortality in patients with ARDS. However, a major limitation of their meta-analysis is that these biomarkers were tested in univariate analyses without considering confounders as disease severity scores. Given the high univariate ORs as compared to the multivariate ORs found in this systematic review, the performance of these biomarkers is likely to be overestimated. Jabaudon et al. [109] found in an individual patient data meta-analysis that high concentrations of plasma RAGE were associated with 90-day mortality independent of driving pressure or tidal volume. However, they could not correct for disease severity score as these differed between studies. Unfortunately, we were unable to perform a meta-analysis on multivariate data because of heterogeneity of the included studies, as transformation of raw data, biomarker concentration cut-offs, time until outcome, and the variables used in the multivariate analyses varied widely between studies. This could be an incentive to standardize the presentation of ARDS biomarker research in terms of statistics and outcome for future analyses or to make individual patient data accessible.

ARDS biomarkers are presumed to reflect the pathophysiology of ARDS, characterized by alveolar-capillary membrane injury, high permeability alveolar oedema, and migration of inflammatory cells [3]. Previously, Terpstra et al. [19] proposed that biomarkers for ARDS development were correlated with alveolar tissue injury, whereas biomarkers for ARDS mortality correlated more with inflammation. In this systematic review, we found that the majority of biomarkers tested for both ARDS development and mortality were surrogates for inflammation. However, following qualitative inspection, biomarkers for inflammation were not evidently associated with either ARDS development or mortality. In contrast, markers for alveolar epithelial injury (plasma RAGE and SpD) and endothelial permeability (plasma Ang-2) seem to be associated with ARDS development. Therefore, we should consider how we intend to use (a set of) biomarkers in patients with ARDS.

A biomarker for ARDS development should be specific for ARDS, i.e. a biomarker that reflects alveolar injury or alveolar-capillary injury. Half of plasma biomarkers for ARDS development included in this study reflected inflammation. An increase in inflammatory biomarkers is known to correlate with increased disease severity scores [71, 97, 110]. In turn, the majority of studies in this review found significantly higher disease severity scores in the critically ill patients that eventually developed ARDS. Thus, plasma biomarkers for inflammation rather represented an estimation of disease severity and its associated increased risk for the development of ARDS. In addition, biomarkers for inflammation in plasma lack the specificity to diagnose ARDS, as they are unlikely to differentiate sepsis with ARDS from sepsis without ARDS. In contrast, locally sampled biomarkers for inflammation, for example in the alveolar space, could potentially diagnose ARDS [111]. Biomarkers used for ARDS mortality or for the identification of less heterogeneous ARDS phenotypes do not require to be ARDS specific, provided that they adequately predict or stratify patients with ARDS.

The heterogeneity of ARDS has been recognized as a major contributor to the negative randomized controlled trial results among patients with ARDS [11]. Therefore, it is necessary to identify homogeneous ARDS phenotypes that are more likely to respond to an intervention. This is known as predictive enrichment [112]. Previously, patients with ARDS have been successfully stratified based on clinical parameters, such as ARDS risk factor (pulmonary or extra-pulmonary) or PaO_2_/FiO_2_ ratio [113]. ARDS biomarkers could be used to stratify patients with ARDS based on biological or pathophysiological phenotype. For example, trials of novel therapies designed to influence vascular permeability may benefit from preferentially enrolling patients with high Ang-2 concentrations. Recently, clinical parameters have been combined with a set of biomarkers in a retrospective latent class analysis. In three trials, two distinct phenotypes were found: hyperinflammatory and hypoinflammatory ARDS [16, 17]. Patients with the hyperinflammatory phenotype had reduced mortality rate with higher positive end-expiratory pressures and with liberal fluid treatment, whereas the trials themselves found no difference between the entire intervention groups. The next step is to validate the identification of ARDS phenotypes based on latent class analysis in prospective studies. An adequate combination of biomarkers and clinical parameters remains to be established. Until now, there is no list of biomarkers that are associated with ARDS development or mortality independently of clinical parameters. This systematic review may guide the selection of ARDS biomarkers used for predictive enrichment.

This systematic review has limitations. First, the intent of this systematic review was to perform a meta-analysis. However, we decided not to perform a meta-analysis, as the biomarker data handling and outcomes varied widely among studies, and pooling would have resulted in a non-informative estimate [21]. Arguably, this is a positive result, as it refrains us from focusing on the few biomarkers that could be pooled in a meta-analysis and guides us into a direction were multiple biomarkers combined with other parameters are of interest. In a heterogeneous syndrome as ARDS, the one biomarker probably does not exist. Second, the first sampling moment varied between sampling at ICU admission until 72 h following ICU admission. Initially, ARDS is characterized by an exudative phase followed by a second proliferative phase and late fibrotic phase [3]. The moment of sampling likely influences biomarker concentrations, as both alveolar membrane injury and inflammation increase during the exudative phase. This is also seen in six biomarkers that have been measured at separate days, resulting in a significant change in adjusted OR for four biomarkers (Table 4) [61, 98, 104, 105]. Third, the aim of this systematic review was to assess the independent risk effects of biomarkers measured in various bodily fluid compartments. However, the majority of studies assessed biomarkers in plasma. It remains to be answered whether other bodily fluid compartments, for example from the airways and alveolar space themselves, might outperform ARDS biomarkers in plasma, especially for ARDS development. Fourth, all studies found in this systematic review used a clinical definition of ARDS as standard for ARDS diagnosis. Given the poor correlation between a clinical diagnosis and a histopathological diagnosis of ARDS, these studies are diagnosing a very heterogeneous disease syndrome [7–10]. In order to actually evaluate ARDS development, biomarkers should be compared to a histopathological image of DAD, although acquiring histology poses great challenges by itself. Fifth, as only biomarkers assessed in multivariate analyses were included in this study, new promising biomarkers evaluated in univariate analyses were excluded from this study. Lastly, non-significant biomarkers in multivariate analyses were more likely not to be reported, although some studies report non-significant results nonetheless.

## Conclusion

In here, we present a list of biomarkers for ARDS mortality and ARDS development tested in multivariate analyses. In multiple studies that assessed Ang-2 and RAGE, high plasma levels were associated with an increased risk of ARDS development. We did not find a biomarker that independently predicted mortality in all studies that assessed the biomarker. Furthermore, biomarker data reporting and variables used in multivariate analyses differed greatly between studies. Taken together, we should look for a combination of biomarkers and clinical parameters in a structured approach in order to find more homogeneous ARDS phenotypes. This systematic review may guide the selection of ARDS biomarkers for ARDS phenotyping.

## Supplementary information

**Additional file 1.** Literature search.

**Additional file 2.** Quality assessment

## Data Availability

The datasets used during the current study are available from the corresponding author on reasonable request.

## Abbreviations

AECC: American European Consensus Conference
Ang-2: Angiopoeitin-2
ARDS: Acute respiratory distress syndrome
CRP: C-reactive protein
DAD: Diffuse alveolar damage
IL-8: Interleukin-8
NOS: Newcastle-Ottawa Scale
OR: Odds ratio
RAGE: Receptor for advanced glycation end products
SpD: Surfactant protein D
VWF: Von Willebrand factor
