# Supplementary material for: A systematic review of biomarkers multivariately associated with acute respiratory distress syndrome development and mortality
Source: Crit Care. 2020 May 24;24:243. doi: 10.1186/s13054-020-02913-7 (PMC7245629; doi:10.1186/s13054-020-02913-7)
Supplement: Supplementary file 1 — Additional file 1. Literature search. [file 13054_2020_2913_MOESM1_ESM.docx]

**Additional file 1**

**Supplement Literature search**

**Embase.com**

('respiratory distress syndrome'/exp OR ('respiratory distress'/de AND 'acute disease'/de) OR (((acute OR syndrom* OR adult* OR idiopath* ) NEAR/3 respirator* NEAR/3 distress*) OR ards):ab,ti) AND (('biological marker'/de OR marker/de OR 'disease marker'/de OR (marker* OR biomarker*):ab,ti) OR (('diagnostic value'/de OR 'diagnostic accuracy'/de OR 'early diagnosis'/de OR 'prognosis'/de OR 'survival'/exp OR 'mortality'/de OR 'fatality'/de OR 'predictor variable'/de OR 'prediction'/de OR ((diagnos* NEAR/3 (value* OR earl* OR accurac* OR utilit*)) OR prognos* OR surviv* OR mortalit* OR fatal* OR predict* OR recogni*):ab,ti) AND ('blood level'/de OR 'cyclic AMP blood level'/exp OR 'enzyme blood level'/exp OR 'histamine blood level'/exp OR 'protein blood level'/exp OR 'serotonin blood level'/exp OR 'somatostatin blood level'/exp OR blood/de OR 'blood analysis'/de OR 'lung lavage'/de OR 'lavage fluid'/de OR 'bronchoalveolar lavage fluid'/de OR 'expired air'/de OR 'breath analysis'/de OR 'concentration (parameters)'/de OR 'blood sampling'/de OR (((blood OR plasma OR serum OR lavage OR expir* OR air OR exhal* OR breath ) NEAR/3 (level* OR concentration* OR profile* OR evaluat* OR mediator* OR sampl* OR analy* OR test*))):ab,ti))) NOT ([animals]/lim NOT [humans]/lim) NOT ([Conference Abstract]/lim OR [Letter]/lim OR [Note]/lim OR [Editorial]/lim) AND [english]/lim

**Medline Ovid**

(Respiratory Distress Syndrome, Adult/ OR (((acute OR syndrom* OR adult* OR idiopath* ) ADJ3 respirator* ADJ3 distress*) OR ards).ab,ti.) AND ((biomarker/ OR (marker* OR biomarker*).ab,ti.) OR ((Early Diagnosis/ OR prognosis/ OR survival/ OR mortality/ OR mortality.xs. OR Fatal Outcome/ OR ((diagnos* ADJ3 (value* OR earl* OR accurac* OR utilit*)) OR prognos* OR surviv* OR mortalit* OR fatal* OR predict* OR recogni*).ab,ti.) AND (blood.xs. OR blood/ OR exp Bronchoalveolar Lavage/ OR Breath Tests/ OR Blood Specimen Collection/ OR (((blood OR plasma OR serum OR lavage OR expir* OR air OR exhal* OR breath) ADJ3 (level* OR concentration* OR profile* OR evaluat* OR mediator* OR sampl* OR analy* OR test*))).ab,ti.))) NOT (exp animals/ NOT humans/) NOT (letter OR news OR comment OR editorial OR congresses OR abstracts).pt. AND english.la.

**Cochrane CENTRAL**

((((acute OR syndrom* OR adult* OR idiopath* ) NEAR/3 respirator* NEAR/3 distress*) OR ards):ab,ti) AND (((marker* OR biomarker*):ab,ti) OR ((((diagnos* NEAR/3 (value* OR earl* OR accurac* OR utilit*)) OR prognos* OR surviv* OR mortalit* OR fatal* OR predict* OR recogni*):ab,ti) AND ((((blood OR plasma OR serum OR lavage OR expir* OR air OR exhal* OR breath ) NEAR/3 (level* OR concentration* OR profile* OR evaluat* OR mediator* OR sampl* OR analy* OR test*))):ab,ti)))

**Web of science**

TS=(((((acute OR syndrom* OR adult* OR idiopath* ) NEAR/2 respirator* NEAR/2 distress*) OR ards)) AND (((marker* OR biomarker*)) OR ((((diagnos* NEAR/2 (value* OR earl* OR accurac* OR utilit*)) OR prognos* OR surviv* OR mortalit* OR fatal* OR predict* OR recogni*)) AND ((((blood OR plasma OR serum OR lavage OR expir* OR air OR exhal* OR breath ) NEAR/2 (level* OR concentration* OR profile* OR evaluat* OR mediator* OR sampl* OR analy* OR test*)))))) NOT ((animal* OR rat OR rats OR mouse OR mice OR murine OR dog OR dogs OR canine OR cat OR cats OR feline OR rabbit OR cow OR cows OR bovine OR rodent* OR sheep OR ovine OR pig OR swine OR porcine OR veterinar* OR chick* OR zebrafish* OR baboon* OR nonhuman* OR primate* OR cattle* OR goose OR geese OR duck OR macaque* OR avian* OR bird*) NOT (human* OR patient*))) AND DT=(article) AND LA=(english)

**Google scholar**

"acute|adult respiratory distress"|ards marker|markers|biomarker|biomarkers|"blood|plasma|serum|lavage|air|breath level|concentration|profile|evaluation|analysis|test" "diagnostic value|accuracy|utility"|prognosis|survival|mortality|fatal|prediction
