# Supplementary material for: A systematic review of biomarkers multivariately associated with acute respiratory distress syndrome development and mortality
Source: Crit Care. 2020 May 24;24:243. doi: 10.1186/s13054-020-02913-7 (PMC7245629; doi:10.1186/s13054-020-02913-7)
Supplement: Supplementary file 2 — Additional file 2. Quality assessment [file 13054_2020_2913_MOESM2_ESM.docx]

**Additional file 2 Quality assessment**

**Table E1 – Newcastle Ottawa Scale for Development of ARDS**

| **No.** | **Reference** | **Selection - Representativeness** | **Selection - Selection non-exposed cohort** | **Selection - Ascertainment of exposure** | **Selection - Outcome not present** | **Comparability - matched for** | **Outcome - Assessment of outcome** | **Outcome - Follow-up duration** | **Outcome - Follow up adequacy** | **Total Score** |
| --- | --- | --- | --- | --- | --- | --- | --- | --- | --- | --- |
| 1 | Agrawal 2013[1] | * | * | * | - | ** | * | * | * | 8 |
| 2 | Ahasic 2012[2] | * | - | * | - | ** | * | * | * | 7 |
| 3 | Aisiku 2016[3] | - | * | * | - | ** | * | * | * | 7 |
| 4 | Amat 2000[4] | * | * | * | - | * | * | * | - | 6 |
| 5 | Bai 2017[5] | - | * | * | - | ** | * | * | * | 7 |
| 5 | Bai 2017[5] | - | * | * | - | ** | * | * | * | 7 |
| 6 | Bai 2018[6] | * | * | * | - | * | * | * | * | 7 |
| 7 | Chen 2019[7] | * | * | - | - | ** | * | - | * | 6 |
| 8 | Du 2016[8] | * | * | * | * | * | * | * | * | 8 |
| 9 | Faust 2020[9] | * | * | * | - | ** | * | * | * | 8 |
| 9 | Faust 2020[9] | * | * | * | - | ** | * | * | * | 8 |
| 10 | Fremont 2010[10] | * | * | * | - | * | * | * | * | 7 |
| 11 | Gaudet 2018[11] | * | * | * | - | * | * | * | - | 6 |
| 12 | Hendrickson 2018[12] | * | * | * | - | ** | * | * | - | 7 |
| 13 | Huang 2019[13] | * | * | * | - | ** | * | * | * | 8 |
| 14 | Huang 2019[14] | - | * | * | - | ** | * | * | * | 7 |
| 15 | Jabaudon 2018[15] | * | * | * | - | ** | * | * | * | 8 |
| 16 | Jensen 2016[16] | * | * | * | - | ** | * | * | * | 8 |
| 17 | Jones 2020[17] | * | * | * | - | ** | * | * | - | 7 |
| 17 | Jones 2020[17] | * | - | - | - | ** | * | * | - | 6 |
| 18 | Komiya 2011[18] | * | - | - | - | * | * | - | * | 4 |
| 19 | Lee 2011[19] | * | * | * | - | * | * | * | - | 6 |
| 20 | Lin 2017[20] | * | * | * | - | ** | * | * | * | 8 |
| 21 | Liu 2017[21] | * | * | * | - | ** | * | * | - | 7 |
| 22 | Luo 2017[22] | * | * | * | * | ** | * | * | * | 9 |
| 23 | Meyer 2017[23] | * | * | * | - | ** | * | * | * | 8 |
| 24 | Mikkelsen 2012[24] | * | - | * | - | ** | * | * | * | 7 |
| 25 | Osaka 2011[25] | - | * | * | - | * | * | * | * | 6 |
| 26 | Palakshappa 2016[26] | * | * | * | - | ** | * | * | * | 8 |
| 27 | Reilly 2018[27] | * | * | * | - | ** | * | * | - | 7 |
| 28 | Shashaty 2019[28] | * | - | * | - | ** | * | * | - | 6 |
| 28 | Shashaty 2019[28] | * | - | * | - | ** | * | * | - | 6 |
| 29 | Shaver 2017[29] | * | * | * | * | ** | * | * | * | 9 |
| 30 | Suzuki 2017[30] | - | * | * | - | * | * | * | * | 6 |
| 31 | Wang 2019[31] | * | * | * | - | ** | * | * | - | 7 |
| 32 | Ware 2017[32] | * | * | * | * | * | * | * | - | 7 |
| 33 | Xu 2018[33] | * | * | * | - | ** | * | * | * | 8 |
| 34 | Yeh 2017[34] | * | * | * | - | ** | * | * | * | 8 |
| 35 | Ying 2019[35] | * | * | * | - | ** | * | * | * | 8 |

Selection representativeness: patients without pre-defined subgroups; selection non-exposed cohort: cohort of patients at-risk for ARDS without selection out of a larger cohort; ascertainment of exposure: cohort study (not case-control); outcome not present: PaO_2_/FiO_2_ ratio presented; comparability: ** variables in multivariate analysis described, * multivariate analysis without description of variables; follow-up duration: at least 7 days, follow-up adequacy: reported no loss to follow-up or missing biomarker values.

**Table E2 – Newcastle Ottawa Scale for Mortality of ARDS**

| **No.** | **Reference** | **Selection - Representativeness** | **Selection - Selection non-exposed cohort** | **Selection - Ascertainment of exposure** | **Selection - Outcome not present** | **Comparability - matched for** | **Outcome - Assessment of outcome** | **Outcome - Follow-up duration** | **Outcome - Follow up adequacy** | **Total Score** |
| --- | --- | --- | --- | --- | --- | --- | --- | --- | --- | --- |
| 1 | Adamzik 2013[36] | * | * | * | * | ** | * | * | * | 9 |
| 2 | Ahasic 2012[2] | * | * | - | * | * | * | * | * | 7 |
| 3 | Amat 2000[4] | * | * | * | * | * | * | * | - | 7 |
| 4 | Bajwa 2008[37] | * | * | - | * | ** | * | * | - | 8 |
| 5 | Bajwa 2009[38] | * | * | - | * | ** | * | * | - | 8 |
| 6 | Bajwa 2013[39] | * | * | * | * | ** | - | * | - | 7 |
| 7 | Calfee 2008[40] | * | * | * | * | ** | - | * | - | 7 |
| 8 | Calfee 2009[41] | * | * | * | * | ** | * | * | - | 8 |
| 9 | Calfee 2011[42] | * | * | * | * | ** | * | * | - | 8 |
| 10 | Calfee 2012[43] | - | * | * | * | ** | * | * | - | 7 |
| 11 | Calfee 2015[44]  Single centre | * | * | * | * | ** | * | - | - | 7 |
| 11 | Calfee 2015[44]  Multi centre | * | * | * | * | ** | * | * | - | 8 |
| 12 | Cartin-Ceba 2015[45] | * | * | * | * | ** | * | - | - | 7 |
| 13 | Chen 2009[46] Validation cohort | * | * | * | * | ** | * | * | * | 9 |
| 14 | Clark 1995[47] | * | * | * | * | ** | * | - | - | 7 |
| 15 | Clark 2013[48] | * | * | * | * | * | * | * | - | 7 |
| 16 | Dolinay 2012[49] | * | - | - | * | ** | * | - | * | 6 |
| 17 | Eisner 2003[50] | * | * | * | * | ** | * | * | - | 8 |
| 18 | Forel 2015[51] | * | * | * | * | ** | * | - | * | 8 |
| **19** | Forel 2018[52] | * | * | - | * | ** | * | * | * | 8 |
| 20 | Guervilly 2011[53] | * | * | * | * | * | * | * | * | 8 |
| 21 | Kim 2019[54] | * | * | * | * | ** | * | * | * | 9 |
| 22 | Lee 2019[55] | * | * | - | * | ** | * | * | * | 8 |
| 23 | Lesur 2006[56] | * | * | * | * | ** | * | * | * | 9 |
| 24 | Li 2018[57] | * | * | * | * | ** | * | * | - | 8 |
| 25 | Lin 2010[58] | * | * | - | * | ** | * | * | * | 8 |
| 26 | Lin 2012[59] | * | * | * | * | ** | * | * | * | 9 |
| 27 | Lin 2013[60] | * | * | * | * | ** | * | * | * | 9 |
| 28 | Madtes 1998[61] | * | * | * | * | * | * | - | - | 6 |
| 29 | McClintock 2006[62] | * | * | - | * | * | * | - | - | 5 |
| 30 | McClintock 2007[63] | * | * | * | * | ** | * | - | - | 7 |
| 31 | McClintock 2008[64] | * | * | * | * | ** | * | - | * | 8 |
| 32 | Menk 2018[65] | * | * | * | * | ** | * | - | - | 7 |
| 33 | Metkus 2017[66] | * | * | * | * | * | * | * | - | 7 |
| 34 | Mrozek 2016[67] | * | * | * | * | ** | * | * | * | 9 |
| 35 | Ong 2010[68] | - | * | * | * | ** | * | * | * | 8 |
| 36 | Parsons 2005[69] | * | * | - | * | ** | * | * | - | 7 |
| 37 | Parsons 2005[70] | * | * | * | * | ** | * | * | - | 8 |
| 38 | Quesnel 2012[71] | * | * | * | * | ** | * | * | * | 9 |
| 39 | Rahmel 2018[72] | * | * | * | * | ** | * | * | * | 9 |
| 40 | Reddy 2019[73] | * | * | * | * | * | * | * | * | 8 |
| 41 | Rivara 2012[74] | * | * | - | * | ** | * | * | * | 8 |
| 42 | Rogers 2019[75] | * | - | * | * | ** | * | * | - | 7 |
| 43 | Sapru 2015[76] | * | * | - | * | ** | * | * | - | 7 |
| 44 | Suratt 2009[77] | * | * | * | * | * | * | - | - | 6 |
| 45 | Tang 2014[78] | * | * | * | * | ** | * | - | * | 8 |
| 46 | Tsangaris 2009[79] | * | * | - | * | ** | * | * | * | 8 |
| 47 | Tsangaris 2017[80] | - | * | - | * | ** | * | * | * | 7 |
| 48 | Tsantes 2013[81] | * | * | * | * | ** | * | * | * | 9 |
| 49 | Tseng 2014[82] | * | * | * | * | ** | * | - | * | 8 |
| 50 | Wang 2017[83] | * | * | * | * | ** | * | * | * | 9 |
| 51 | Wang 2018[84] | * | * | * | * | * | * | - | * | 7 |
| 52 | Ware 2004[85] | * | * | * | * | ** | * | - | - | 7 |
| 53 | Xu 2017[86] | * | * | * | * | ** | * | * | * | 9 |

Selection representativeness: patients with ARDS without pre-defined subgroups; selection non-exposed cohort: cohort of patients with ARDS without selection out of a larger cohort; ascertainment of exposure: PaO_2_/FiO_2_ ratio presented; outcome not present: patient being alive upon inclusion; comparability: ** variables in multivariate analysis described, * multivariate analysis without description of variables; follow-up duration: at least 28 days, follow-up adequacy: reported no loss to follow-up or missing biomarker values.

**References:**

1. Agrawal A, Matthay MA, Kangelaris KN, Stein J, Chu JC, Imp BM, Cortez A, Abbott J, Liu KD, Calfee CS: **Plasma angiopoietin-2 predicts the onset of acute lung injury in critically ill patients**. *American journal of respiratory and critical care medicine* 2013, **187**(7):736-742.

2. Ahasic AM, Zhai R, Su L, Zhao Y, Aronis KN, Thompson BT, Mantzoros CS, Christiani DC: **IGF1 and IGFBP3 in acute respiratory distress syndrome**. *Eur J Endocrinol* 2012, **166**(1):121-129.

3. Aisiku IP, Yamal JM, Doshi P, Benoit JS, Gopinath S, Goodman JC, Robertson CS: **Plasma cytokines IL-6, IL-8, and IL-10 are associated with the development of acute respiratory distress syndrome in patients with severe traumatic brain injury**. *Crit Care* 2016, **20**(1).

4. Amat M, Barcons M, Mancebo J, Mateo J, Oliver A, Mayoral JF, Fontcuberta J, Vila L: **Evolution of leukotriene B4, peptide leukotrienes, and interleukin-8 plasma concentrations in patients at risk of acute respiratory distress syndrome and with acute respiratory distress syndrome: Mortality prognostic study**. *Critical care medicine* 2000, **28**(1):57-62.

5. Bai W, Zhu WL, Ning YL, Li P, Zhao Y, Yang N, Chen X, Jiang YL, Yang WQ, Jiang DP *et al*: **Dramatic increases in blood glutamate concentrations are closely related to traumatic brain injury-induced acute lung injury**. *Scientific reports* 2017, **7**(1):5380.

6. Bai W, Li W, Ning YL, Li P, Zhao Y, Yang N, Jiang YL, Liang ZP, Jiang DP, Wang Y *et al*: **Blood glutamate levels are closely related to acute lung injury and prognosis after stroke**. *Front Neurol* 2018, **8**(JAN).

7. Chen D, Wu X, Yang J, Yu L: **Serum plasminogen activator urokinase receptor predicts elevated risk of acute respiratory distress syndrome in patients with sepsis and is positively associated with disease severity, inflammation and mortality**. *Exp Ther Med* 2019, **18**(4):2984-2992.

8. Du SL, Ai J, Zeng XZ, Wan J, Wu X, He JX: **Plasma level of advanced oxidation protein products as a novel biomarker of acute lung injury following cardiac surgery**. *Springerplus* 2016, **5**.

9. Faust HE, Reilly JP, Anderson BJ, Ittner CAG, Forker CM, Zhang P, Weaver BA, Holena DN, Lanken PN, Christie JD *et al*: **Plasma Mitochondrial DNA Levels Are Associated With ARDS in Trauma and Sepsis Patients**. *Chest* 2020, **157**(1):67-76.

10. Fremont RD, Koyama T, Calfee CS, Wu W, Dossett LA, Bossert FR, Mitchell D, Wickersham N, Bernard GR, Matthay MA *et al*: **Acute lung injury in patients with traumatic injuries: Utility of a panel of biomarkers for diagnosis and pathogenesis**. *J Trauma Inj Infect Crit Care* 2010, **68**(5):1121-1127.

11. Gaudet A, Parmentier E, Dubucquoi S, Poissy J, Duburcq T, Lassalle P, De Freitas Caires N, Mathieu D: **Low endocan levels are predictive of Acute Respiratory Distress Syndrome in severe sepsis and septic shock**. *Journal of critical care* 2018, **47**:121-126.

12. Hendrickson CM, Gibb SL, Miyazawa BY, Keating SM, Ross E, Conroy AS, Calfee CS, Pati S, Cohen MJ: **Elevated plasma levels of TIMP-3 are associated with a higher risk of acute respiratory distress syndrome and death following severe isolated traumatic brain injury**. *Trauma surg acute care open* 2018, **3**(1):e000171.

13. Huang X, Zhao M: **High expression of long non-coding RNA MALAT1 correlates with raised acute respiratory distress syndrome risk, disease severity, and increased mortality in sepstic patients**. *Int J Clin Exp Pathol* 2019, **12**(5):1877-1887.

14. Huang Y, Xiao J, Cai T, Yang L, Shi F, Wang Y, Li Y, Shi T, Li C, Peng Y *et al*: **Immature granulocytes: A novel biomarker of acute respiratory distress syndrome in patients with acute pancreatitis**. *Journal of critical care* 2019, **50**:303-308.

15. Jabaudon M, Berthelin P, Pranal T, Roszyk L, Godet T, Faure JS, Chabanne R, Eisenmann N, Lautrette A, Belville C *et al*: **Receptor for advanced glycation end-products and ARDS prediction: a multicentre observational study**. *Sci rep* 2018, **8**(1):2603.

16. Jensen JUS, Itenov TS, Thormar KM, Hein L, Mohr TT, Andersen MH, Løken J, Tousi H, Lundgren B, Boesen HC *et al*: **Prediction of non-recovery from ventilator-demanding acute respiratory failure, ARDS and death using lung damage biomarkers: data from a 1200-patient critical care randomized trial**. *Ann Intensive Care* 2016, **6**(1).

17. Jones TK, Feng R, Kerchberger VE, Reilly JP, Anderson BJ, Shashaty MGS, Wang F, Dunn TG, Riley TR, Abbott J *et al*: **Plasma sRAGE acts as a genetically regulated causal intermediate in sepsis-associated acute respiratory distress syndrome**. *American journal of respiratory and critical care medicine* 2020, **201**(1):47-56.

18. Komiya K, Ishii H, Teramoto S, Takahashi O, Eshima N, Yamaguchi O, Ebi N, Murakami J, Yamamoto H, Kadota JI: **Diagnostic utility of C-reactive Protein combined with brain natriuretic peptide in acute pulmonary edema: A cross sectional study**. *Respiratory research* 2011, **12**(1).

19. Lee KA, Gong MN: **Pre-B-cell colony-enhancing factor and its clinical correlates with acute lung injury and sepsis**. *Chest* 2011, **140**(2):382-390.

20. Lin J, Zhang W, Wang L, Tian F: **Diagnostic and prognostic values of Club cell protein 16 (CC16) in critical care patients with acute respiratory distress syndrome**. *J Clin Lab Anal* 2017.

21. Liu XW, Ma T, Cai Q, Wang L, Song HW, Liu Z: **Elevation of Serum PARK7 and IL-8 Levels Is Associated With Acute Lung Injury in Patients With Severe Sepsis/Septic Shock**. *J Intensive Care Med* 2017.

22. Luo J, Yu H, Hu YH, Liu D, Wang YW, Wang MY, Liang BM, Liang ZA: **Early identification of patients at risk for acute respiratory distress syndrome among severe pneumonia: A retrospective cohort study**. *J Thorac Dis* 2017, **9**(10):3979-3995.

23. Meyer NJ, Reilly JP, Feng R, Christie JD, Hazen SL, Albert CJ, Franke JD, Hartman CL, McHowat J, Ford DA: **Myeloperoxidase-derived 2-chlorofatty acids contribute to human sepsis mortality via acute respiratory distress syndrome**. *JCI insight* 2017, **2**(23).

24. Mikkelsen ME, Shah CV, Scherpereel A, Lanken PN, Lassalle P, Bellamy SL, Localio AR, Albelda SM, Meyer NJ, Christie JD: **Lower serum endocan levels are associated with the development of acute lung injury after major trauma**. *J Crit Care* 2012, **27**(5).

25. Osaka D, Shibata Y, Kanouchi K, Nishiwaki M, Kimura T, Kishi H, Abe S, Inoue S, Tokairin Y, Igarashi A *et al*: **Soluble endothelial selectin in acute lung injury complicated by severe pneumonia**. *Int J Med Sci* 2011, **8**(4):302-308.

26. Palakshappa JA, Anderson BJ, Reilly JP, Shashaty MGS, Ueno R, Wu Q, Ittner CAG, Tommasini A, Dunn TG, Charles D *et al*: **Low plasma levels of adiponectin do not explain acute respiratory distress syndrome risk: A prospective cohort study of patients with severe sepsis**. *Crit Care* 2016, **20**(1).

27. Reilly JP, Wang F, Jones TK, Palakshappa JA, Anderson BJ, Shashaty MGS, Dunn TG, Johansson ED, Riley TR, Lim B *et al*: **Plasma angiopoietin-2 as a potential causal marker in sepsis-associated ARDS development: evidence from Mendelian randomization and mediation analysis**. *Intensive care medicine* 2018, **44**(11):1849-1858.

28. Shashaty MGS, Reilly JP, Faust HE, Forker CM, Ittner CAG, Zhang PX, Hotz MJ, Fitzgerald D, Yang W, Anderson BJ *et al*: **Plasma receptor interacting protein kinase-3 levels are associated with acute respiratory distress syndrome in sepsis and trauma: A cohort study**. *Crit Care* 2019, **23**(1).

29. Shaver CM, Woods J, Clune JK, Grove BS, Wickersham NE, McNeil JB, Shemancik G, Ware LB, Bastarache JA: **Circulating microparticle levels are reduced in patients with ARDS**. *Crit Care* 2017, **21**(1).

30. Suzuki A, Taniguchi H, Kondoh Y, Ando M, Watanabe N, Kimura T, Kataoka K, Yokoyama T, Sakamoto K, Hasegawa Y: **Soluble thrombomodulin in bronchoalveolar lavage fluid is an independent predictor of severe drug-induced lung injury**. *Respirology* 2017, **22**(4):744-749.

31. Wang Y, Fu X, Yu B, Ai F: **Long non-coding RNA THRIL predicts increased acute respiratory distress syndrome risk and positively correlates with disease severity, inflammation, and mortality in sepsis patients**. *J Clin Lab Anal* 2019, **33**(6).

32. Ware LB, Zhao Z, Koyama T, Brown RM, Semler MW, Janz DR, May AK, Fremont RD, Matthay MA, Cohen MJ *et al*: **Derivation and validation of a two-biomarker panel for diagnosis of ARDS in patients with severe traumatic injuries**. *Trauma surg acute care open* 2017, **2**(1):e000121.

33. Xu Z, Wu GM, Li Q, Ji FY, Shi Z, Guo H, Yin JB, Zhou J, Gong L, Mei CX *et al*: **Predictive Value of Combined LIPS and ANG-2 Level in Critically Ill Patients with ARDS Risk Factors**. *Mediators of inflammation* 2018, **2018**:1739615.

34. Yeh LC, Huang PW, Hsieh KH, Wang CH, Kao YK, Lin TH, Lee XL: **Elevated plasma levels of Gas6 are associated with acute lung injury in patients with severe sepsis**. *Tohoku J Exp Med* 2017, **243**(3):187-193.

35. Ying J, Zhou D, Gu T, Huang J: **Endocan, a Risk Factor for Developing Acute Respiratory Distress Syndrome among Severe Pneumonia Patients**. *Can Respir J* 2019, **2019**.

36. Adamzik M, Broll J, Steinmann J, Westendorf AM, Rehfeld I, Kreissig C, Peters J: **An increased alveolar CD4 + CD25 + Foxp3 + T-regulatory cell ratio in acute respiratory distress syndrome is associated with increased 30-day mortality**. *Intensive care medicine* 2013, **39**(10):1743-1751.

37. Bajwa EK, Januzzi JL, Gong MN, Thompson BT, Christiani DC: **Prognostic value of plasma N-terminal probrain natriuretic peptide levels in the acute respiratory distress syndrome**. *Critical care medicine* 2008, **36**(8):2322-2327.

38. Bajwa EK, Khan UA, Januzzi JL, Gong MN, Thompson BT, Christiani DC: **Plasma C-reactive protein levels are associated with improved outcome in ARDS**. *Chest* 2009, **136**(2):471-480.

39. Bajwa EK, Volk JA, Christiani DC, Harris RS, Matthay MA, Thompson BT, Januzzi JL: **Prognostic and diagnostic value of plasma soluble suppression of tumorigenicity-2 concentrations in acute respiratory distress syndrome**. *Critical care medicine* 2013, **41**(11):2521-2531.

40. Calfee CS, Ware LB, Eisner MD, Parsons PE, Thompson BT, Wickersham N, Matthay MA: **Plasma receptor for advanced glycation end products and clinical outcomes in acute lung injury**. *Thorax* 2008, **63**(12):1083-1089.

41. Calfee CS, Eisner MD, Parsons PE, Thompson BT, Conner Jr ER, Matthay MA, Ware LB: **Soluble intercellular adhesion molecule-1 and clinical outcomes in patients with acute lung injury**. *Intensive care medicine* 2009, **35**(2):248-257.

42. Calfee CS, Ware LB, Glidden DV, Eisner MD, Parsons PE, Thompson BT, Matthay MA: **Use of risk reclassification with multiple biomarkers improves mortality prediction in acute lung injury**. *Critical care medicine* 2011, **39**(4):711-717.

43. Calfee CS, Gallagher D, Abbott J, Thompson BT, Matthay MA: **Plasma angiopoietin-2 in clinical acute lung injury: Prognostic and pathogenetic significance**. *Critical care medicine* 2012, **40**(6):1731-1737.

44. Calfee CS, Janz DR, Bernard GR, May AK, Kangelaris KN, Matthay MA, Ware LB: **Distinct molecular phenotypes of direct vs indirect ARDS in single-center and multicenter studies**. *Chest* 2015, **147**(6):1539-1548.

45. Cartin-Ceba R, Hubmayr RD, Qin R, Peters S, Determann RM, Schultz MJ, Gajic O: **Predictive value of plasma biomarkers for mortality and organ failure development in patients with acute respiratory distress syndrome**. *Journal of critical care* 2015, **30**(1):219.e211-219.e217.

46. Chen CY, Yang KY, Chen MY, Chen HY, Lin MT, Lee YC, Perng RP, Hsieh SL, Yang PC, Chou TY: **Decoy receptor 3 levels in peripheral blood predict outcomes of acute respiratory distress syndrome**. *American journal of respiratory and critical care medicine* 2009, **180**(8):751-760.

47. Clark JG, Milberg JA, Steinberg KP, Hudson LD: **Type III procollagen peptide in the adult respiratory distress syndrome. Association of increased peptide levels in bronchoalveolar lavage fluid with increased risk for death**. *Annals of internal medicine* 1995, **122**(1):17-23.

48. Clark BJ, Bull TM, Benson AB, Stream AR, Macht M, Gaydos J, Meadows C, Burnham EL, Moss M: **Growth differentiation factor-15 and prognosis in acute respiratory distress syndrome: A retrospective cohort study**. *Crit Care* 2013, **17**(3).

49. Dolinay T, Kim YS, Howrylak J, Hunninghake GM, An CH, Fredenburgh L, Massaro AF, Rogers A, Gazourian L, Nakahira K *et al*: **Inflammasome-regulated cytokines are critical mediators of acute lung injury**. *American journal of respiratory and critical care medicine* 2012, **185**(11):1225-1234.

50. Eisner MD, Parsons P, Matthay MA, Ware L, Greene K: **Plasma surfactant protein levels and clinical outcomes in patients with acute lung injury**. *Thorax* 2003, **58**(11):983-988.

51. Forel JM, Guervilly C, Hraiech S, Voillet F, Thomas G, Somma C, Secq V, Farnarier C, Payan MJ, Donati SY *et al*: **Type III procollagen is a reliable marker of ARDS-associated lung fibroproliferation**. *Intensive care medicine* 2015, **41**(1):1-11.

52. Forel JM, Guervilly C, Farnarier C, Donati SY, Hraiech S, Persico N, Allardet-Servent J, Coiffard B, Gainnier M, Loundou A *et al*: **Transforming growth factor-β1 in predicting early lung fibroproliferation in patients with acute respiratory distress syndrome**. *PloS one* 2018, **13**(11).

53. Guervilly C, Lacroix R, Forel JM, Roch A, Camoin-Jau L, Papazian L, Dignat-George F: **High levels of circulating leukocyte microparticles are associated with better outcome in acute respiratory distress syndrome**. *Crit Care* 2011, **15**(1).

54. Kim JM, Lee JK, Choi SM, Lee J, Park YS, Lee CH, Yim JJ, Yoo CG, Kim YW, Han SK *et al*: **Diagnostic and prognostic values of serum activin-a levels in patients with acute respiratory distress syndrome**. *BMC pulmonary medicine* 2019, **19**(1).

55. Lee HW, Choi SM, Lee J, Park YS, Lee CH, Yim JJ, Yoo CG, Kim YW, Han SK, Lee SM: **Serum Uric Acid Level as a Prognostic Marker in Patients With Acute Respiratory Distress Syndrome**. *J Intensive Care Med* 2019, **34**(5):404-410.

56. Lesur O, Langevin S, Berthiaume Y, Légaré M, Skrobik Y, Bellemare JF, Lévy B, Fortier Y, Lauzier F, Bravo G *et al*: **Outcome value of Clara cell protein in serum of patients with acute respiratory distress syndrome**. *Intensive care medicine* 2006, **32**(8):1167-1174.

57. Li W, Ai X, Ni Y, Ye Z, Liang Z: **The Association Between the Neutrophil-to-Lymphocyte Ratio and Mortality in Patients With Acute Respiratory Distress Syndrome: A Retrospective Cohort Study**. *Shock (Augusta, Ga)* 2019, **51**(2):161-167.

58. Lin MT, Wei YF, Ku SC, Lin CA, Ho CC, Yu CJ: **Serum soluble triggering receptor expressed on myeloid cells-1 in acute respiratory distress syndrome: A prospective observational cohort study**. *J Formos Med Assoc* 2010, **109**(11):800-809.

59. Lin Q, Fu F, Chen H, Zhu B: **Copeptin in the assessment of acute lung injury and cardiogenic pulmonary edema**. *Respiratory medicine* 2012, **106**(9):1268-1277.

60. Lin Q, Shen J, Shen L, Zhang Z, Fu F: **Increased plasma levels of heparin-binding protein in patients with acute respiratory distress syndrome**. *Crit Care* 2013, **17**(4).

61. Madtes DK, Rubenfeld G, Klima LD, Milberg JA, Steinberg KP, Martin TR, Raghu G, Hudson LD, Clark JG: **Elevated transforming growth factor-α levels in bronchoalveolar lavage fluid of patients with acute respiratory distress syndrome**. *American journal of respiratory and critical care medicine* 1998, **158**(2):424-430.

62. McClintock DE, Starcher B, Eisner MD, Thompson BT, Hayden DL, Church GD, Matthay MA, Wiedemann HP, Arroliga AC, Fisher Jr CJ *et al*: **Higher urine desmosine levels are associated with mortality in patients with acute lung injury**. *American journal of physiology Lung cellular and molecular physiology* 2006, **291**(4):L566-L571.

63. McClintock DE, Ware LB, Eisner MD, Wickersham N, Thompson BT, Matthay MA, Wiedemann HP, Arroliga AC, Fisher Jr CJ, Komara Jr JJ *et al*: **Higher urine nitric oxide is associated with improved outcomes in patients with acute lung injury**. *American journal of respiratory and critical care medicine* 2007, **175**(3):256-262.

64. McClintock D, Zhuo H, Wickersham N, Matthay MA, Ware LB: **Biomarkers of inflammation, coagulation and fibrinolysis predict mortality in acute lung injury**. *Crit Care* 2008, **12**(2):R41.

65. Menk M, Giebelhäuser L, Vorderwülbecke G, Gassner M, Graw JA, Weiss B, Zimmermann M, Wernecke KD, Weber-Carstens S: **Nucleated red blood cells as predictors of mortality in patients with acute respiratory distress syndrome (ARDS): an observational study**. *Ann Intensive Care* 2018, **8**(1).

66. Metkus TS, Guallar E, Sokoll L, Morrow D, Tomaselli G, Brower R, Schulman S, Korley FK: **Prevalence and prognostic association of circulating troponin in the acute respiratory distress syndrome**. *Critical care medicine* 2017, **45**(10):1709-1717.

67. Mrozek S, Jabaudon M, Jaber S, Paugam-Burtz C, Lefrant JY, Rouby JJ, Asehnoune K, Allaouchiche B, Baldesi O, Leone M *et al*: **Elevated Plasma Levels of sRAGE Are Associated With Nonfocal CT-Based Lung Imaging in Patients With ARDS: A Prospective Multicenter Study**. *Chest* 2016, **150**(5):998-1007.

68. Ong T, McClintock DE, Kallet RH, Ware LB, Matthay MA, Liu KD: **Ratio of angiopoietin-2 to angiopoietin-1 as a predictor of mortality in acute lung injury patients**. *Critical care medicine* 2010, **38**(9):1845-1851.

69. Parsons PE, Matthay MA, Ware LB, Eisner MD: **Elevated plasma levels of soluble TNF receptors are associated with morbidity and mortality in patients with acute lung injury**. *American journal of physiology Lung cellular and molecular physiology* 2005, **288**(3 32-3):L426-L431.

70. Parsons PE, Eisner MD, Thompson BT, Matthay MA, Ancukiewicz M, Bernard GR, Wheeler AP: **Lower tidal volume ventilation and plasma cytokine markers of inflammation in patients with acute lung injury**. *Critical care medicine* 2005, **33**(1):1-6.

71. Quesnel C, Piednoir P, Gelly J, Nardelli L, Garnier M, Leçon V, Lasocki S, Bouadma L, Philip I, Elbim C *et al*: **Alveolar fibrocyte percentage is an independent predictor of poor outcome in Patients with acute lung injury**. *Critical care medicine* 2012, **40**(1):21-28.

72. Rahmel T, Rump K, Adamzik M, Peters J, Frey UH: **Increased circulating microRNA-122 is associated with mortality and acute liver injury in the acute respiratory distress syndrome**. *BMC anesthesiology* 2018, **18**(1).

73. Reddy R, Asante I, Liu S, Parikh P, Liebler J, Borok Z, Rodgers K, Baydur A, Louie SG: **Circulating angiotensin peptides levels in Acute Respiratory Distress Syndrome correlate with clinical outcomes: A pilot study**. *PloS one* 2019, **14**(3).

74. Rivara MB, Bajwa EK, Januzzi JL, Gong MN, Thompson BT, Christiani DC: **Prognostic significance of elevated cardiac troponin-T levels in acute respiratory distress syndrome patients**. *PloS one* 2012, **7**(7).

75. Rogers AJ, Guan J, Trtchounian A, Hunninghake GM, Kaimal R, Desai M, Kozikowski LA, DeSouza L, Mogan S, Liu KD *et al*: **Association of Elevated Plasma Interleukin-18 Level With Increased Mortality in a Clinical Trial of Statin Treatment for Acute Respiratory Distress Syndrome**. *Critical care medicine* 2019, **47**(8):1089‐1096.

76. Sapru A, Calfee CS, Liu KD, Kangelaris K, Hansen H, Pawlikowska L, Ware LB, Alkhouli MF, Abbot J, Matthay MA: **Plasma soluble thrombomodulin levels are associated with mortality in the acute respiratory distress syndrome**. *Intensive care medicine* 2015, **41**(3):470-478.

77. Suratt BT, Eisner MD, Calfee CS, Allard JB, Whittaker LA, Engelken DT, Petty JM, Trimarchi T, Gauthier L, Parsons PE: **Plasma granulocyte colony-stimulating factor levels correlate with clinical outcomes in patients with acute lung injury**. *Critical care medicine* 2009, **37**(4):1322-1328.

78. Tang L, Zhao Y, Wang D, Deng W, Li C, Li Q, Huang S, Shu C: **Endocan levels in peripheral blood predict outcomes of acute respiratory distress syndrome**. *Mediators of inflammation* 2014, **2014**.

79. Tsangaris I, Tsantes A, Bonovas S, Lignos M, Kopterides P, Gialeraki A, Rapti E, Orfanos S, Dimopoulou I, Travlou A *et al*: **The impact of the PAI-1 4G/5G polymorphism on the outcome of patients with ALI/ARDS**. *Thromb Res* 2009, **123**(6):832-836.

80. Tsangaris I, Tsantes A, Vrigkou E, Kopterides P, Pelekanou A, Zerva K, Antonakos G, Konstantonis D, Mavrou I, Tsaknis G *et al*: **Angiopoietin-2 Levels as Predictors of Outcome in Mechanically Ventilated Patients with Acute Respiratory Distress Syndrome**. *Dis Markers* 2017, **2017**.

81. Tsantes AE, Kopterides P, Bonovas S, Bagos P, Antonakos G, Nikolopoulos GK, Gialeraki A, Kapsimali V, Kyriakou E, Kokori S *et al*: **Effect of angiotensin converting enzyme gene I/D polymorphism and its expression on clinical outcome in acute respiratory distress syndrome**. *Minerva anestesiologica* 2013, **79**(8):861-870.

82. Tseng CC, Fang WF, Leung SY, Chen HC, Chang YC, Wang CC, Chang HC, Lin MC: **Impact of serum biomarkers and clinical factors on intensive care unit mortality and 6-month outcome in relatively healthy patients with severe pneumonia and acute respiratory distress syndrome**. *Dis Markers* 2014, **2014**.

83. Wang T, Zhu Z, Liu Z, Yi L, Yang Z, Bian W, Chen W, Wang S, Li G, Li A *et al*: **Plasma Neutrophil Elastase and Elafin as Prognostic Biomarker for Acute Respiratory Distress Syndrome: A Multicenter Survival and Longitudinal Prospective Observation Study**. *Shock (Augusta, Ga)* 2017, **48**(2):168-174.

84. Wang Y, Ju M, Chen C, Yang D, Hou D, Tang X, Zhu X, Zhang D, Wang L, Ji S *et al*: **Neutrophil-to-lymphocyte ratio as a prognostic marker in acute respiratory distress syndrome patients: A retrospective study**. *J Thorac Dis* 2018, **10**(1):273-282.

85. Ware LB, Eisner MD, Thompson BT, Parsons PE, Matthay MA: **Significance of Von Willebrand factor in septic and nonseptic patients with acute lung injury**. *American journal of respiratory and critical care medicine* 2004, **170**(7):766-772.

86. Xu Z, Li X, Huang Y, Mao P, Wu S, Yang B, Yang Y, Chen K, Liu X, Li Y: **The predictive value of plasma galectin-3 for ards severity and clinical outcome**. *Shock (Augusta, Ga)* 2017, **47**(3):331-336.
